# Using ESTIMATE algorithm to establish an 8-mRNA signature prognosis prediction system and identify immunocyte infiltration-related genes in Pancreatic adenocarcinoma

**DOI:** 10.18632/aging.102931

**Published:** 2020-03-17

**Authors:** Zibo Meng, Dianyun Ren, Kun Zhang, Jingyuan Zhao, Xin Jin, Heshui Wu

**Affiliations:** 1 Department of Pancreatic Surgery, Union Hospital, Tongji Medical College, Huazhong University of Science and Technology, Wuhan 430022, China; 2 Sino-German Laboratory of Personalized Medicine for Pancreatic Cancer, Union Hospital, Tongji Medical College, Huazhong University of Science and Technology, Wuhan 430022, China; 3 Department of Otorhinolaryngology-Head And Neck Surgery, Union Hospital, Tongji Medical College, Huazhong University of Science and Technology, Wuhan 430022, China; 4 Cancer Center, Union Hospital, Tongji Medical College, Huazhong University of Science and Technology, Wuhan 430022, China

**Keywords:** pancreatic cancer, tumor microenvironment, immunocytes infiltration, FOXO1

## Abstract

Objective: The tumour microenvironment is one of the significant factors driving the carcinogenesis of Pancreatic adenocarcinoma (PAAD). However, the underlying mechanism of how the tumour microenvironment impacts the prognosis of PAAD is not completely clear.

Results: The transcriptome and clinical data of 182 PAAD program cases were downloaded from the TCGA database. Three hundred thirty-three differentially expressed genes (DEGs) between high and low stromal groups and 314 DEGs between high and low immune score groups were identified using ESTIMATE score. Based on the 203 genes differentially expressed simultaneously in two score-related comparisons, we established an 8-mRNA signature to evaluate the prognosis of PAAD patients. Kaplan-Meier curves showed significantly worse survival for patients with high-risk scores in both the training and validation groups. The risk score was an independent prognostic factor and had a high predictive value for the prognosis of patients with PAAD. By searching the TCGA database, we showed that CA9, CXCL9, and GIMAP7 from the 8-mRNA signature were associated with the infiltration levels of immunocytes by regulating FOXO1 expression in PAAD.

Conclusions: Unlike traditional methods of screening for differential genes in cancer and healthy tissues, we constructed a novel 8-mRNA signature to predict the prognosis of PAAD patients by applying ESTIMATE scoring to RNA-seq-based transcriptome data. Most importantly, we identified CA9, CXCL9, and GIMAP7 from the above eight genes as regulators of immunocyte infiltration by adjusting the expression of FOXO1 in PAAD. Thus, CA9, CXCL9, and GIMAP7 might be the ideal targets of immune therapy of PAAD.

Methods: ESTIMATE scoring was used to determine the stromal and immune scores of transcriptome datasets downloaded from the TCGA database. An mRNA-based prognostic signature was built for the training cohort via the LASSO Cox regression model. The signature was verified using a validation cohort. Kaplan-Meier curves and log-rank analysis were used to identify survival differences. Western blot analysis and RT-qPCR analysis were carried out to analyze the expression of specific proteins and mRNAs. IHC was performed to assess the protein levels of Forkhead box-O 1 (FOXO1), Carbonic anhydrase 9 (CA9), C-X-C motif chemokine ligand 9 (CXCL9), and GTPase, IMAP family member 7 (GIMAP7) in the tissue microarray of PAAD.

## INTRODUCTION

Pancreatic adenocarcinoma (PAAD) is one of the most devastating human malignant tumours in the world [1]. Because of its resistance to chemoradiotherapy and the trend of early metastasis, the 5-year survival rate of PAAD is less than 5% [2, 3]. Approximately 39,590 patients die of this disease in the United States yearly [4]. Besides, the morbidity and mortality rates of PAAD are ninth and sixth, respectively, among Malignant tumours in China, which shows a rapid growth pattern that matches the trend in other countries [5]. Thus, a better understanding of the pathogenesis of cancer and the exploration of more therapeutic strategies is imperative for improving the prognosis of PAAD patients.

Malignant solid tumour tissues consist of tumour cells, tumour-associated normal epithelial, vascular cells, immune cells, and stromal cells [6]. Cancer-associated fibroblasts, adipocytes, pericytes, mesenchymal stem cells, endothelial cells, lymphocytes, and extracellular matrix are components of a tumour microenvironment [7]. PAAD is characterized by an intense stromal desmoplastic reaction around cancer cells [8], which plays an essential role in tumorigenesis and drug resistance [9]. Therefore, the tumour microenvironment is one of the significant factors determining the prognosis of PAAD. However, the mechanism of how the tumour microenvironment engineers the carcinogenesis of PAAD is not entirely clear.

A tumour microenvironment consists of multiple sorts of inflammatory cells and mediators that modulate tumour development, growth, and metastasis [10]. Tumour infiltrated immunocytes encompass macrophages, dendritic cells (DCs), myeloid-derived suppressor cells (MDSCs), and T lymphocytes [10]. Immunocytes in a tumour microenvironment produce a variety of proinflammatory cytokines to maintain chronic inflammation and regulate tumour growth and progression [11]. On the contrary, immunosuppressive Tregs and MDSCs suppress T lymphocyte proliferation [12]. Changes in the proportion and function of different types of tumour-infiltrating immunocytes contribute to PAAD initiation and progression [11, 12]**.** However, the mechanism regulating the infiltration of immunocytes in the tumour environment of PAAD is poorly understood.

Since immunocytes and stromal cells represent significant components of the tumour microenvironment in PAAD [13, 14], we first calculated the stromal and immune scores of PAAD using ‘Estimation of Stromal and Immune cells in Malignant Tumours using Expression data’ (ESTIMATE) scoring system to assess the level of infiltrating stromal and immune cells in the tumour microenvironment [15]. Our research using bioinformatics analysis explored differentially expressed genes (DEGs) related to stromal and immune scores in the tumour microenvironment, and we identified eight genes that contributed to the overall survival rate of PAAD. Moreover, we found that three of the eight genes (CA9, CXCL9, and GIMAP7) associated independently with the overall survival rate and have close relationships with immunocyte infiltration in PAAD. Furthermore, we revealed that FOXO1 potentially serves as the downstream target of these three genes in modulating immunocyte infiltration in the tumour microenvironment of PAAD. In summary, our study provided an 8-mRNA signature system to predict the prognosis of PAAD and identified three genes (CA9, CXCL9, and GIMAP7) that were associated with immunocyte infiltration. Most importantly, CA9, CXCL9, and GIMAP7 could serve as the target of immune therapy of PAAD.

## RESULTS

### The ESTIMATE algorithm identifies DEGs associated with stroma and immune scores

**Figure 1:**
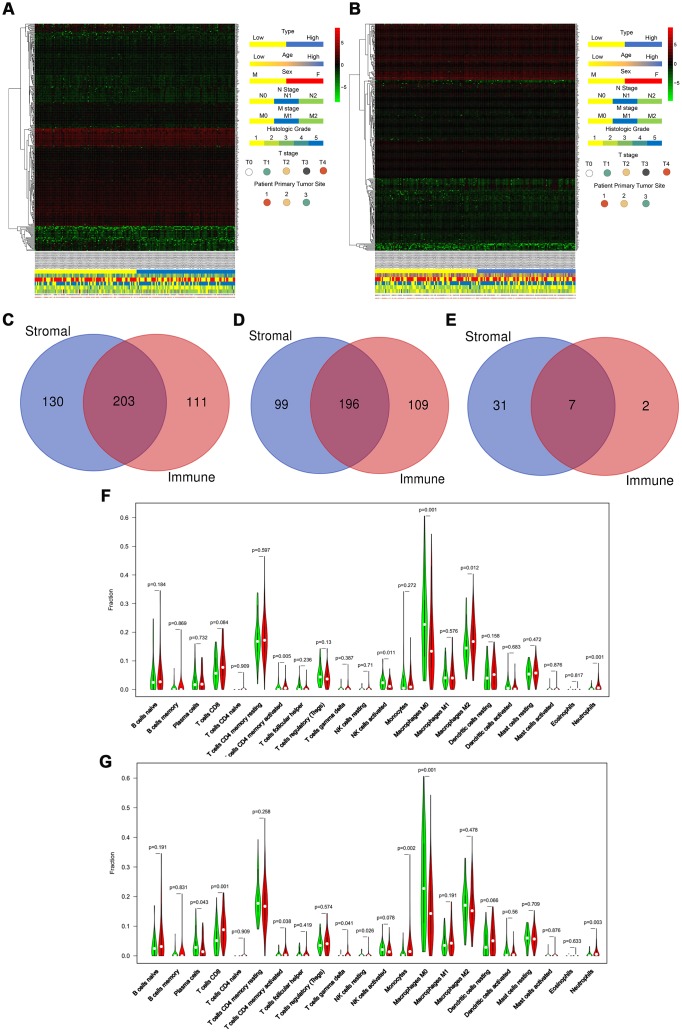
**ESTIMATE algorithm identifies DEGs associated with stroma and immune scores.** (**A** and **B**) Heatmaps displayed distinct mRNA expression forms and clinicopathological features between high and low stromal score groups (**A**) and between high and low immune score groups (**B**). (Primary tumor site: 1 for pancreas head, 2 for pancreas body, and 3 for other locations) (**C**–**E**) The Venn diagram showed the simultaneously differentially expressed DEGs (**C**), the simultaneously upregulated differentially expressed DEGs (**D**), and the simultaneously downregulated differentially expressed DEGs (**E**) between stromal score and immune score groups. (**F**, **G**) CIBERSORT results showed the association between the infiltration levels of immune cells and the stromal-score level (**F**) and the immune-score level (**G**).

We downloaded the transcriptome and clinical data of 182 PAAD program cases from the TCGA database. Based on the ESTIMATE database results and the median values of the stromal and immune scores (excluding the normal pancreas cases), we divided these patients into high and low stromal score groups and high and low immune score groups. We displayed distinct these clinicopathological features, including age, sex, N stage, M stage, histologic grade, T stage, and primary tumor sites, and mRNA expression forms between high and low stromal (Figure 1A) and between high and low immune (Figure 1B) score groups in the form of heatmaps. We found statistically significant results only for sex and M stage between high and low stromal scores (P = 0.010 and P = 0.010, respectively), whereas only for N stage between high and low immune scores (P = 0.049) as shown in Table 1. And the age, M stage, histologic grade, T stage, and primary tumor sites were not significantly different between two groups. Low stromal scores group was characterized by a higher number of male patients and poorly M stages compared with high stromal scores group. Low immune scores group included more patients with better N stages compared with high immune scores group. Based on our threshold value (log fold change ≥1.5 and P <0.05), there were 333 DEGs between high and low stromal groups and 314 DEGs between high and low immune score groups (Supplementary Tables 5 and 6). The Venn diagram (Figure 1C) used to find the co-DEGs in the two groups showed that 203 genes, which accounted for the majority of DEGs, were differentially expressed simultaneously in two score-related comparisons. Moreover, these simultaneously upregulated and downregulated differentially expressed DEGs were 196 and 7, respectively, with the same alternative trend between the two groups (Figure 1D and 1E). As shown in Figure 1F and 1G, CIBERSORT results revealed that CD4+ activated memory T cells (P = 0.005), M2 macrophages (P = 0.012), and neutrophils (P<0.001) were upregulated, while activated NK cells (P = 0.011) and M0 macrophages (P = 0.001) were downregulated in the high stromal-score group. In the high immune-score group, CD8 T cells (P = 0.001), gamma delta T cell (P = 0.041), resting NK cells (P=0.026), monocytes (P = 0.002), and neutrophils (P = 0.003) were upregulated, while plasma cells (P = 0.043) and M0 macrophages (P = 0.001) were downregulated. Hence, we used the ESTIMATE algorithm to identify DEGs associated with stroma and immune scores successfully.

**Table 1:** The clinicopathological characteristics based on low/high score groups in the training set.

**TCGA cohort**						
**Variables (%)**	**Groups according to stromal score**			**Groups according to Immune score**		
	**Low score (n=89)**	**High score (n=87)**	**P**	**Low score (n=89)**	**High score (n=87)**	**P**
Age(mean, IQR)	64.5(56.0-74.0)	64.8(58.0-73.0)	0.888	65.0(57.0-73.5)	64.3(56.0-73.0)	0.689
Gender			0.010			0.099
Female	32(36.0)	48(55.2)		35(39.3)	45(51.7)	
Male	57(64.0)	39(44.8)		54(60.7)	42(48.3)	
Race			0.819			0.643
White	79(88.8)	76(87.4)		77(86.5)	78(89.7)	
Other races	10(11.2)	11(12.6)		12(13.5)	9(10.3)	
Primary tumor site			0.135			
Pancreas head	65(73.0)	72(82.8)		63(70.8)	74(85.1)	
Pancreas body	19(21.3)	9(10.3)		21(23.6)	7(8.0)	
Other locations	5(5.0)	6(6.9)		5(5.6)	6(6.9)	
T stage			0.423			0.423
T1	5(5.6)	2(2.3)		5(5.6)	2(2.3)	
T2	13(14.6)	11(12.6)		13(14.6)	11(12.6)	
T3	68(76.4)	72(82.8)		68(76.4)	72(82.8)	
T4	1(1.1)	2(2.3)		1(1.1)	2(2.3)	
TX	2(2.2)	0(0.0)		2(2.2)	0(0.0)	
N stage			0.192			0.049
N0	28(31.5)	21(24.1)		27(30.3)	22(25.3)	
N1	57(64.0)	65(74.7)		57(64.0)	65(74.7)	
NX	4(4.5)	1(1.1)		5(5.6)	0(0.0)	
M stage			0.010			0.085
M0	30(33.7)	49(56.3)		33(37.1)	46(52.9)	
M1	2(2.2)	2(2.3)		3(3.4)	1(1.1)	
MX	57(64.0)	36(41.4)		53(59.6)	40(46.0)	
Grade			0.469			0.329
G1	18(20.2)	12(13.8)		19(21.3)	11(12.6)	
G2	44(49.4)	50(57.5)		45(50.6)	49(56.3)	
G3	24(27.0)	24(27.6)		22(24.7)	26(29.9)	
G4	1(1.1)	1(1.1)		1(1.1)	1(1.1)	
GX	2(2.2)	0(0.0)		2(2.2)	0(0.0)	

### KEGG and GO functional enrichment analysis for DEGs

**Figure 2:**
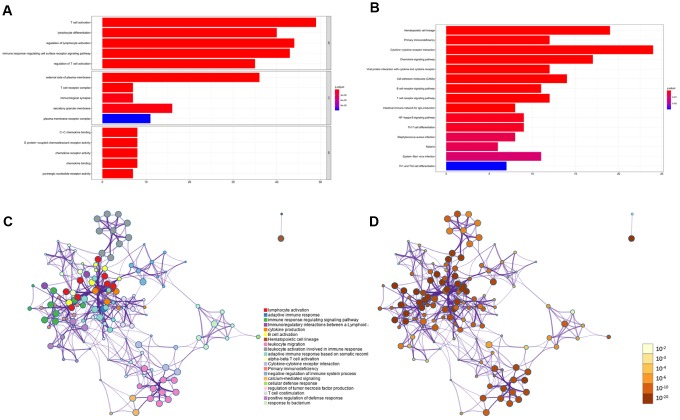
**KEGG and GO functional enrichment analysis for DEGs.** (**A**) Heatmap exhibited the top 15 enriched GO terms across the DEGs. (**B**) Heatmap exhibited the top 15 enriched KEGG pathways across the DEGs. (**C**–**D**) Protein-protein interaction networks from the DEGs which coloured by different cluster ID (**C**) and coloured by p-value (**D**).

To identify differentially regulated genes on immune cell infiltration, we used Metascape tools to conduct the GO and KEGG analyses of the 203 simultaneously expressed DEGs. The Heatmap in Figure 2A exhibits the top 15 enriched GO terms across the DEGs based on Biological Process (BP), Cellular Component (CC), and Molecular Function (MF), and the heatmap in Figure 2B also exhibits the top 15 enriched KEGG pathways across the DEGs. Among them, most terms or pathways were associated with biological processes and lymphocyte activation. We also applied protein-protein interaction networks from the DEGs, which were coloured by different cluster IDs (Figure 2C) and P-values (Figure 2D), and the networks contained more genes that tended to have more significant p-values. Since most networks from the DEGs were related to biological processes and lymphocyte activation, the result confirmed in reverse that the DEGs identified based on stroma and immune scores were accurate. Consequently, combined with the result showed in Figure 1, our study identified that most of the DEGs identified based on the immune and stromal scores were correlated considerably with the immune responses or immune cell infiltration in PAAD.

### An 8-mRNA signature system was established to predict the overall survival of PAAD patients

**Figure 3:**
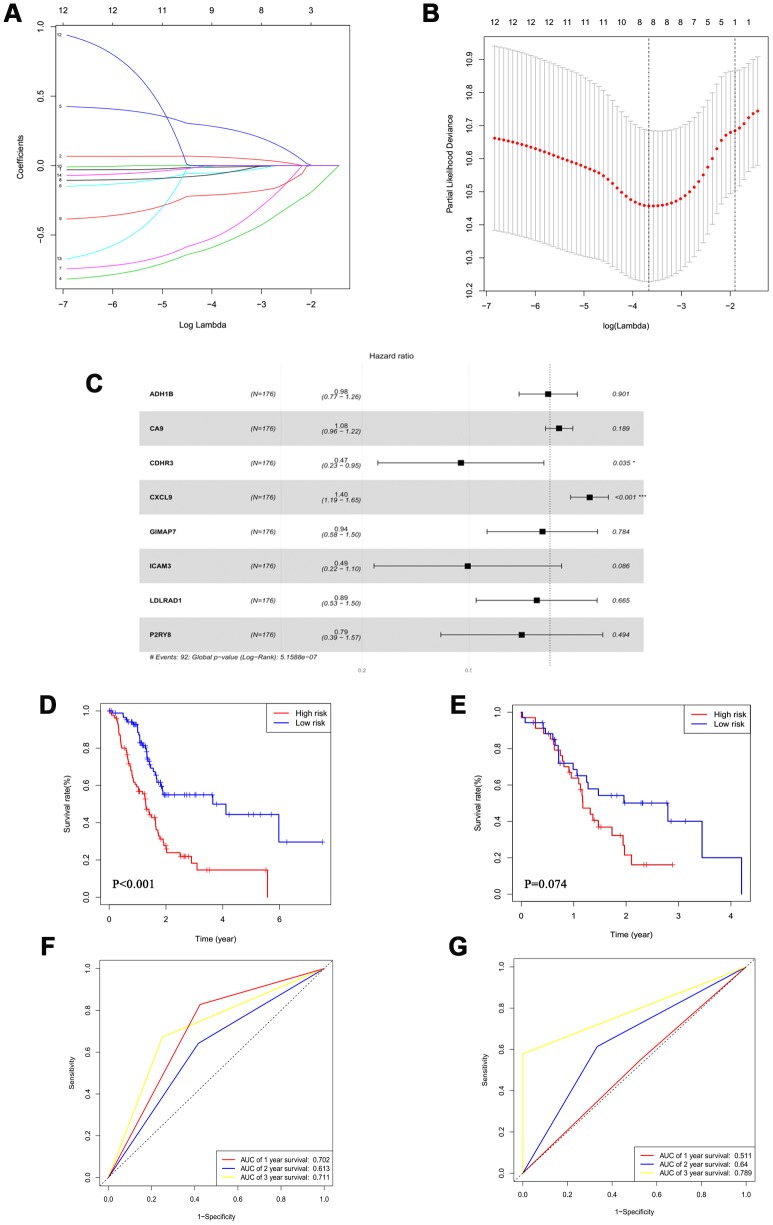
**An 8-mRNA signature system was established to predict the overall survival of PAAD patients.** (**A**) LASSO coefficient profiles of 14 mRNA with P<0.01. (**B**) 10-fold cross-validations result which identified optimal values of the penalty parameter λ. (**C**) The association between each gene and overall survival. (**D**, **E**) The Kaplan-Meier curves in the training set (**D**) and the validation set (**E**). (**F**, **G**) Time-dependent ROC analysis at 1, 2 and 3 years in the training set (**F**) and the validation set (**G**).

Based on the 203 DEGs obtained from the mRNA difference analysis (Figure 1C), we first conducted Cox univariate regression for initial screening to remove interferences from excessive confounding genes and acquire genes with the most significant impact on prognosis. As shown in Supplementary Table 1, to avoid exclusion of important prognostic genes, 14 of the mRNA with P <0.1 were then moved into LASSO regression [16, 17]. We presented the LASSO coefficient profiles of the 14 mRNA (Figure 3A) and produced 10-fold cross-validation results that identified optimal values of the penalty parameter λ (Figure 3B).

The forest plot demonstrated the association between each gene and overall survival (Figure 3C). According to these results, we identified an 8-mRNA signature to evaluate the overall survival time of PAAD patients based on the expression of the 8 mRNAs and their regression coefficients as follows: Risk score = (-0.01579 × expression level of ADH1B) + (0.07838 × expression level of CA9) + (-0.76361 × expression level of CDHR3) + (0.33812 × expression level of CXCL9) + (-0.06606 × expression level of GIMAP7) + (-0.70384 × expression level of ICAM3) + (-0.11457 × expression level of LDLRAD1) + (-0.24179 × expression level of P2RY8). Patients in the TCGA cohort were divided into a low-risk group (N=88) and a high-risk group (N=88) utilizing the median risk score as the cut-off value.

The Kaplan-Meier curves showed that high-risk patients had significantly worse survival rates in the training set (P<0.001) (Figure 3D and Supplementary Figure 2A). Moreover, the multivariate analysis revealed that this prognostic signature was an independent prognostic factor in PAAD patients (Table 2).

**Table 2:** The univariate and multivariate analysis of prognostic factors in pancreatic cancer patients.

**TCGA cohort**				
**Variables**	**Univariate analysis**		**Multivariate analysis**	
	**HR(95%CI)**	**P**	**HR(95%CI)**	**P**
Risk score	1.86(1.57-2.20)	<0.001	1.93(1.60-2.34)	<0.001
Age	1.03(1.01-1.05)	0.007		
Tumor location				
Head	1		1	
Body or tail	0.58(0.31-1.06)	0.077	0.51(0.31-0.82)	0.006
Other location	0.11(0.02-0.76)	0.025		
Gender				
Female	1			
Male	0.82(0.54-1.24)	0.343		
Race				
White	1			
Others	0.89(0.49-1.64)	0.709		
Stromal score				
Low stromal score	1			
High stromal score	1.07(0.71-1.62)	0.743		
Immune score				
Low immune score	1			
High immune score	0.99(0.65-1.49)	0.945		
Tumor stage				
I	1			
II	2.33(1.07-5.09)	0.033		
III	1.25(0.15-10.26)	0.834		
IV	2.11(0.43-10.28)	0.357		
Grade				
G1	1			
G2	1.98(1.02-3.86)	0.043		
G3	2.62(1.30-5.28)	0.007		
G4	1.65(0.21-12.87)	0.634		

We conducted a time-dependent ROC analysis at one, two, and three years to assess the prognostic accuracy of the risk score (Figure 3F and Supplementary Figure 2C). We used 69 patients from ICGC PACA-Australia as the validation cohort and calculated the risk score of each case based on our 8-mRNA signature. Per the Kaplan-Meier curves, the high-risk group patients had worse outcomes (Figure 3E and Supplementary Figure 2B); the time-dependent ROC analysis verified that the risk score had a good long-term prognostic accuracy (Figure 3G and Supplementary Figure 2D). We, therefore, successfully established an 8-mRNA signature to assess the prognosis of PAAD patients.

### CA9, CXCL9, and GIMAP7 have a close relationship with the immune infiltration of the tumour microenvironment in PAAD

**Figure 4:**
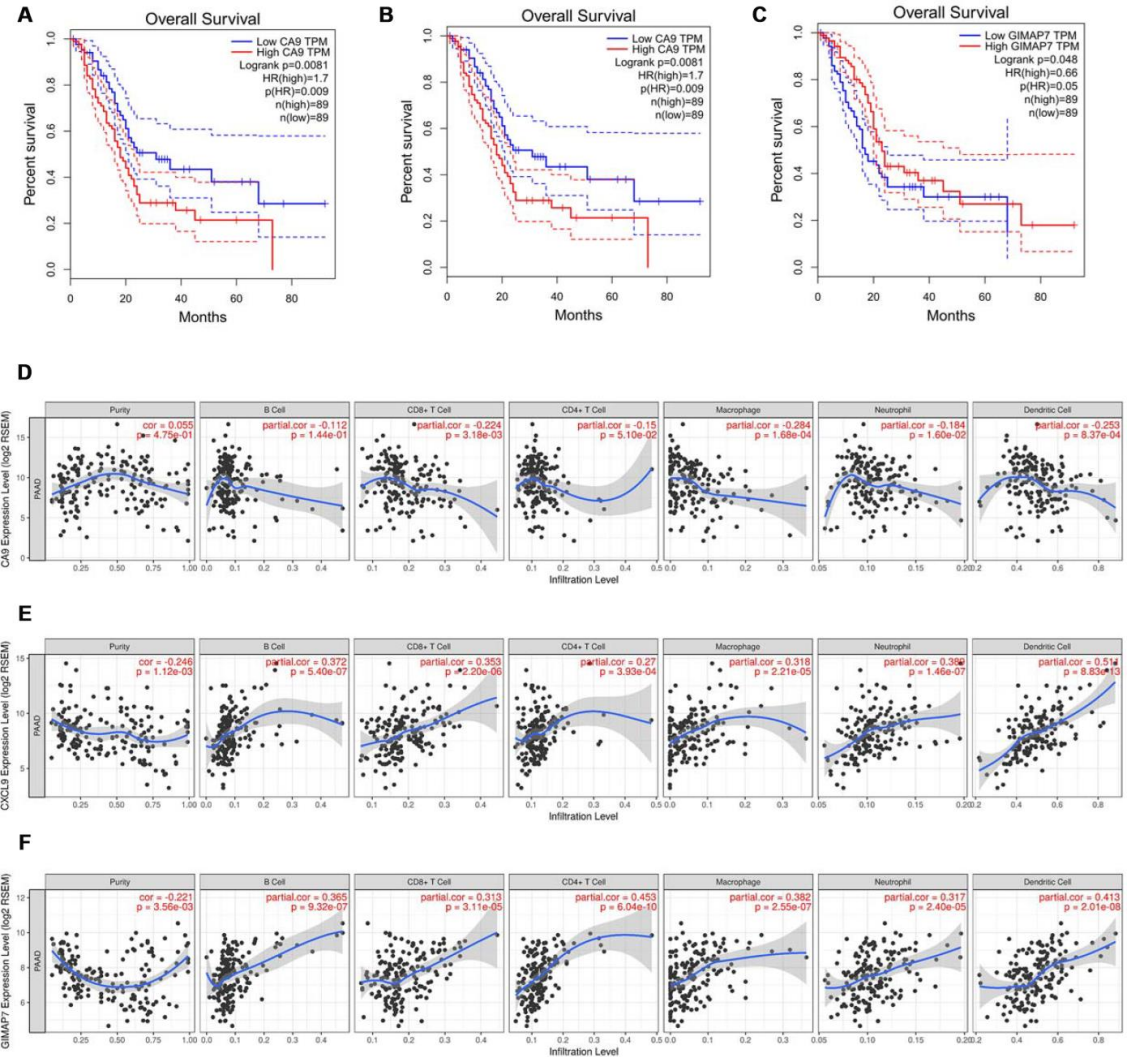
**CA9, CXCL9, and GIMAP7 regulate the immune infiltration of tumour microenvironment in PAAD.** (**A**–**C**) The overall survival rate of the patients with PAAD were computed with the GEPIA web tool. (**D**–**F**) The Timer web tool was used to determine the association between the expression levels of CA9 (**D**), CXCL9 (**E**) and GIMAP7 (**F**) with the infiltration level of immune cells in PAAD samples.

To further explore the regulatory mechanisms for the prognosis of PAAD based on the eight differentially expressed genes in our signature, we searched the GEPIA web tool [18] for an association between the expression of genes at the mRNA level and Overall Survival (OS). The result showed that carbonic anhydrase 9 (CA9), C-X-C motif chemokine ligand 9 (CXCL9), and GTPase, IMAP family member 7 (GIMAP7) were independently associated with the overall survival (OS) rate of PAAD (Figure 4A–4C) but not with the other five factors (Supplementary Figure 1A–1E). So CA9, CXCL9, and GIMAP7 were included for further exploration.

As shown in Figure 1F and 1G, the DEGs were identified based on the immune microenvironment scores, and the GO and KEGG analysis showed that the DEGs correlated significantly with immune reactions or immune cell infiltration in PAAD. So we tried to determine the relationship between the expression levels of these three factors and the immune infiltration level in the tumour microenvironment. The infiltration levels of CD8+ T cells, macrophage cells, neutrophil cells, and dendritic cells were down-regulated by CA9 (Figure 4D). The evaluation of CXCL9 in PAAD showed a positive correlation with infiltration levels of B cells, CD8+ T cells, CD4+ T cells, macrophage cells, neutrophil cells, and dendritic cells (Figure 4E). The analysis of GIAMP7 revealed that the infiltration levels of B cells, CD8+ T cells, CD4+ T cells, macrophage cells, neutrophil cells, and dendritic cells were all up-regulated with the increased expression level of GIMAP7 (Figure 4F). When taken together, these data suggest that CA9, CXCL9, and GIMAP7 contribute to the immune infiltration of the tumour microenvironment in PAAD

### CA9, CXCL9, and GIMAP7 regulate the expression level of FOXO1 in PAAD

**Figure 5:**
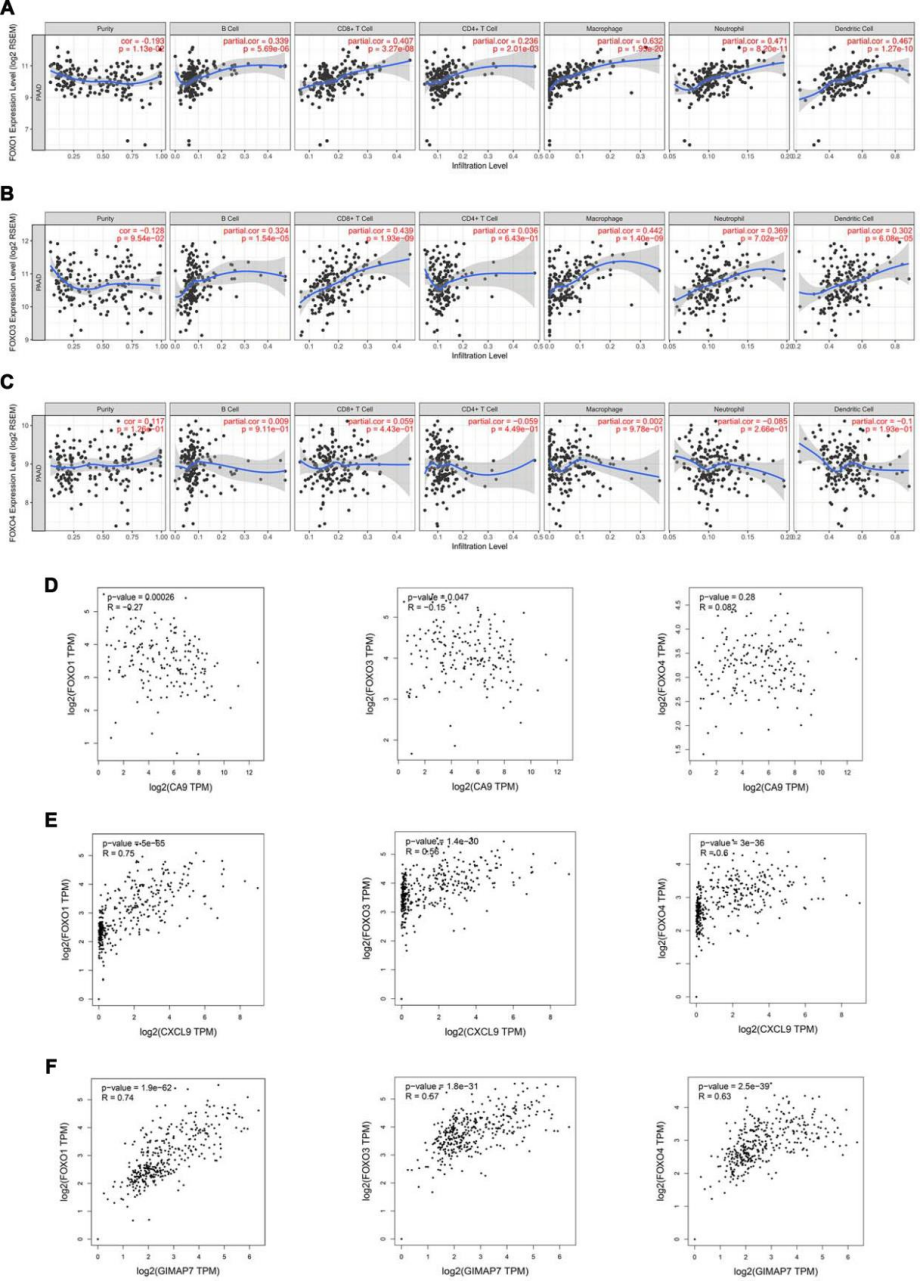
**FOXOs regulate the immune infiltration of tumor microenvironment in PAAD.** (**A**–**C**) The Timer web tool was used to determine the association between the expression levels of FOXO1 (**A**), FOXO3 (**B**), and FOXO4 (**C**) with the infiltration level of immune cells in PAAD samples. (**D**–**F**) The GEPIA web tool was used to determine the correlation between the mRNA expression levels of CA9 (**D**), CXCL9 (**E**), and GIMAP7 (**F**) with FoxOs in PAAD samples, respectively.

Reportedly, the Forkhead box O (FOXOs) family intrinsically influences the anti-tumour immune response and the infiltration levels of immune cells, including CD4+ T cells and CD8+ T cells, B cells, neutrophil cells, macrophage cells, and dendritic cells [19]. Consistent with this report, our data also showed a significant correlation between the expression level of FOXO1 or FOXO3, but not FOXO4, and the infiltration levels of these immune cells (Figure 5A–5C). Furthermore, by searching the GEPIA web tool, we found a negative correlation between the mRNA expression level of CA9 and the FOXOs family in PAAD patients (Figure 5D). On the contrary, the mRNA expression level of CXCL9 correlated positively with FOXOs (Figure 5E), and so did mRNA expression level of GIMAP7 with FOXOs in PAAD (Figure 5F). Consequently, we hypothesised that CA9, CXCL9, and GIMAP7 regulated the infiltration level of immune cells by modifying FOXOs in PAAD.

**Figure 6:**
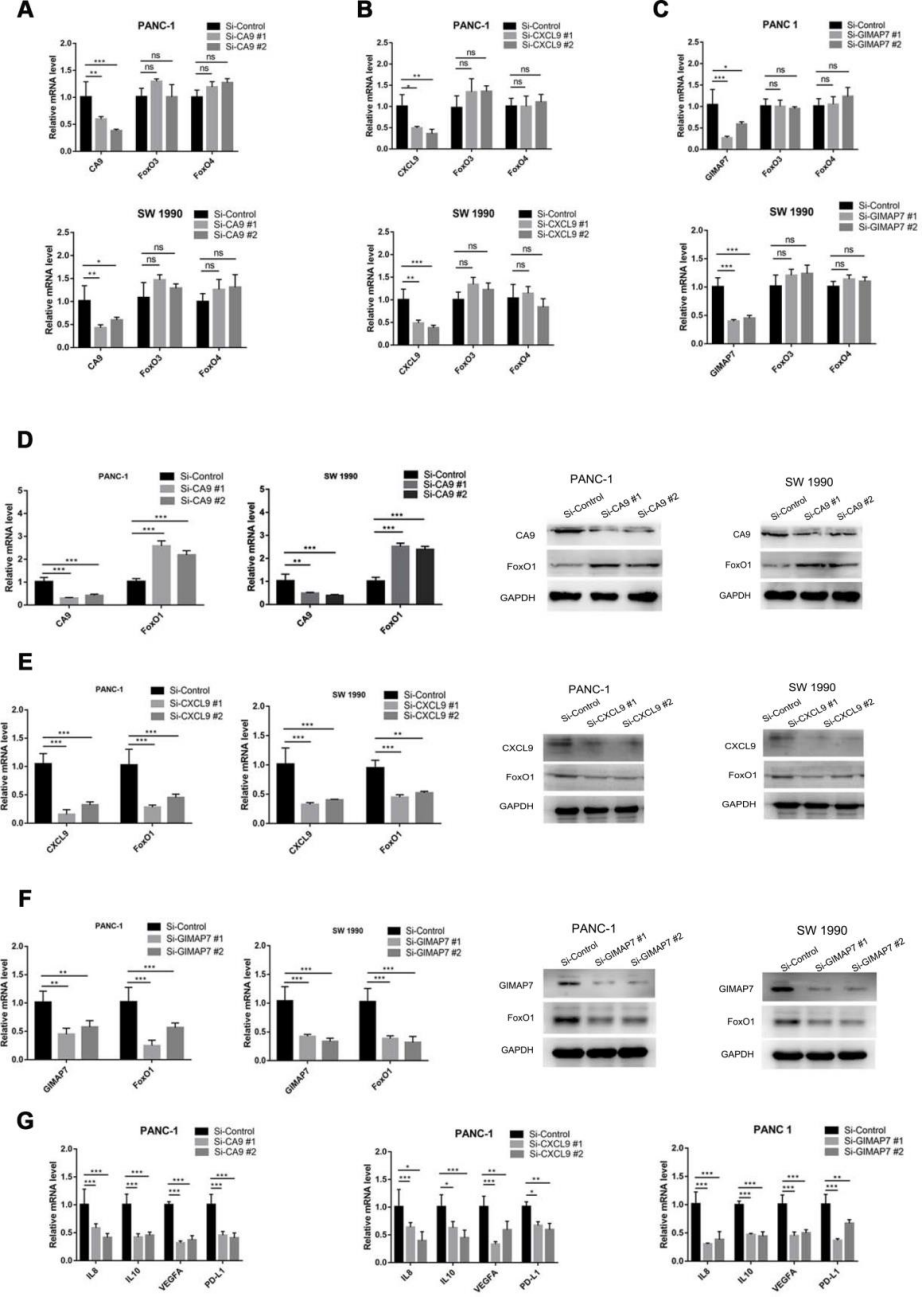
**CA9, CXCL9, and GIMAP7 regulate the expression level of FOXO1 in PAAD.** PANC-1 and SW 1990 cells were transfected with indicated siRNA. Then, 24 hrs post-transfection, cells were harvested for RT-qPCR. The data shown were the mean values ± SD from three replicates. *, P < 0.05; **, P < 0.01; ***, P < 0.001. After 48 h post-transfection, cells were harvested for Western Blot analysis. (**A**–**C**) PANC-1 and SW 1990 cells transfected with Si-CA9 (**A**), Si-CXCL9 (**B**) and Si-GIMAP7 (**C**) were harvested for RT-qPCR. (**D**–**F**) PANC-1 and SW 1990 cells transfected with Si-CA9 (**D**), Si-CXCL9 (**E**) and Si-GIMAP7 (**F**) were harvested for RT-qPCR and western blotting. (**G**) PANC-1 cells transfected with Si-CA9 (**C**), Si-CXCL9 (**E**), or Si-GIMAP7 (**G**) were harvested for RT-qPCR.

To determine whether CA9, CXCL9, and GIMAP7 regulated the immune cell infiltration via FOXOs, we first analyzed the relationship between FOXO3 and FOXO4 and these three genes. We knocked down each of CA9, CXCL9, or GIMAP7 using a gene-specific siRNA and detected the changes in the mRNA level of FOXO3 or FOXO4. We found that these three genes (CA9, CXCL9, or GIMAP7) had apparent effects on FOXO3 and FOXO4 in both PANC-1 and SW 1190 cells (Figure 6A–6C). In contrast, the examination of the correlation between FOXO1 with these three genes indicated that repressing the level of CA9 increased the protein and mRNA levels of FOXO1 in PAAD cells (Figure 6D), whereas, the inhibition of CXCL9 or GIMAP7 by siRNA down-regulated the levels of FOXO1 in PANC-1 and SW 1990 cells (Figure 6E and 6F).

We next examined the downstream inflammatory factors of FOXO1, such as IL-8, IL-10, VEGF, and PD-L1 [20–23] and found that knocking down CA9 significantly decreased the mRNA levels of IL-8, IL-10, VEGF, and PD-L1, but the knockdown of CXCL9 and GIMAP7 substantially increased IL-8, IL-10, VEGF, and PD-L1 expression levels in PAAD cells (Figure 6H and Supplementary Figure 1F). Therefore, our results indicate that CA9 transcriptionally inhibits, while CXCL9 and GIMAP7 transcriptionally promote FOXO1 expression in PAAD.

### CA9, CXCL9, and GIMAP7 correlate with FOXO1 in PAAD patient specimens

**Figure 7:**
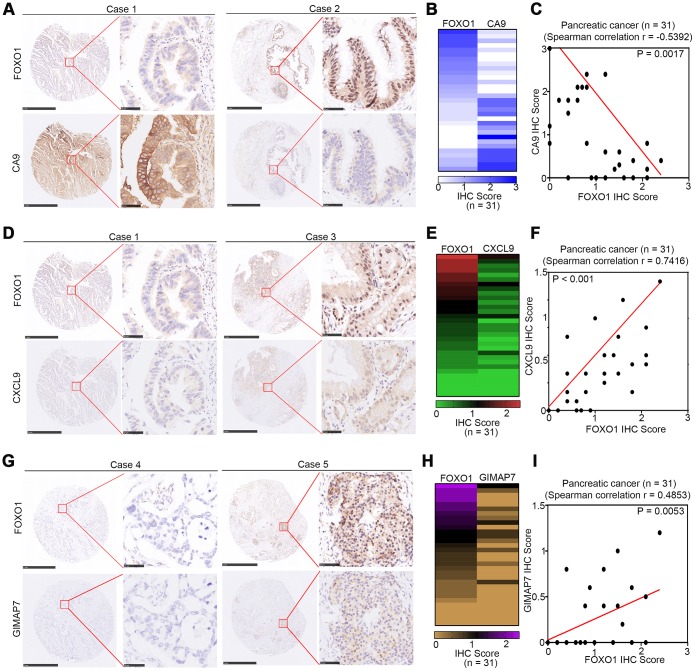
**CA9, CXCL9, and GIMAP7 are correlated with FOXO1 in PAAD patient specimens respectively.** (**A**–**C**) the tissue microarray of PAAD patients (n=31) was stained with FOXO1 and CA9 respectively. The typical image of FOXO1 and CA9 was shown in (**A**), the IHC scores of FOXO1 and CA9 was shown in (**B**) and the correlation of these two proteins was shown in (**C**). (**D**–**F**) the tissue microarray of PAAD patients (n=31) was stained with FOXO1 and CXCL9, respectively. The typical image of FOXO1 and CXCL9 was shown in (**D**), the IHC scores of FOXO1 and CXCL9 was shown in (**E**) and the correlation of these two proteins was shown in (**F**). (**G**–**I**) the tissue microarray of PAAD patients (n=31) was stained with FOXO1 and GIMAP7, respectively. The typical image of FOXO1 and GIMAP7 was shown in (**G**), the IHC scores of FOXO1 and GIMAP7 was shown in (**H**), and the correlation of these two proteins was shown in (**I**). The scale in **A**, **D**, and **G** represents 1mm or 50um, respectively.

To determine the correlation between these three proteins and FOXO1 in PAAD specimens, we examined the expression of all four proteins by immunohistochemistry (IHC) on a TMA containing a PAAD samples cohort (n = 31). Representative images of the high and low/no stainings of CA9, CXCL9, GIMAP7, and FOXO1 are shown in Figure 7A, 7D, and 7G. The IHC score was calculated by multiplying the percentage of positively stained cells with the staining intensity. The expression of CA9 correlated negatively with FOXO1 (Spearman’s product-moment correlation coefficient r = -0.5392, P = 0.0017) (Figure 7B, 7C), which was consistent with the protein and mRNA level changes in pancreatic cell lines reported above. Moreover, our data showed that there was a positive relationship between the expression levels of CXCL9 or GINAP7 and FOXO1 in PAAD patient specimens (Spearman’s product-moment correlation coefficient r = 0.7416, P < 0.001 for CXCL7 and FOXO1, Spearman’s product-moment correlation coefficient r = 0.4853, P = 0.0053 for GIMAP7 and FOXO1) (Figure 7D–7G). Consequently, our results suggest that the expression of CA9, CXCL9, and GIMAP7 all correlated with the expression of FOXO1 in PAAD patient specimens.

## DISCUSSION

Reportedly, ESTIMATE scores could be used to predict cancer patient survival time and evaluate chemotherapeutic drug resistance [15]. Therefore, applying ESTIMATE to RNA-seq-based transcriptome profiles, as well as clinical data, may help to elucidate the facilitating role of the microenvironment to the infiltration of neoplastic cells and provide new insights into the context in which genomic alterations occur. Stromal and immune cells from the tumour microenvironment play an essential role in tumour initiation and progression and are associated with the prognosis of cancer patients [24]. Since a cancerous pancreas has a high stromal score compared with a healthy pancreas, the ESTIMATE score can be applied for the assessment of the infiltration of immune cells and the presence of stromal cells in tumour samples [25, 26]. In our study, we identified 333 DEGs between high and low stromal groups and 314 DEGs between high and low immune score groups. Among all these genes DEGs, 203 genes were differentially expressed simultaneously in two score-related comparisons.

Several studies have reported signatures that could effectively predict overall patient survival, including a five-miRNA signature [27], a 3-lncRNA signature [28], and a specific four genes signature [29]. However, the differentially expressed molecules in these studies were all conducted based on the expression-related survival risk score, and, due to the heterogeneity among different patients, many genes involved in the development of cancer were ignored. Therefore, we have adopted a new patient grouping method based on the different immune microenvironment scores to explore DEGs.

Based on material from the TCGA database, we obtained a unique 8-mRNA signature from the 203 simultaneously expressed DEGs for the prediction of the overall survival of PAAD. Accordingly, this prognostic signature was an independent prognostic factor in PAAD patients, with excellent long-term prognostic accuracy; the high-risk group patients had worse outcomes. By comparing our finding with other models from literature (AUC of the ROC curve varied from 0.62-0.742) [27–29], our model presented more significant advantages in terms of accuracy in predicting prognosis and was promising to be applied for clinical prognostic evaluation of PC patients. Nevertheless, the existence of limitation couldn’t be neglected in our research. The OS and DFS P value of the 8-mRNA model in the validation cohort is not significant as a result of that only 69 patients from ICGC PACA-Australia, which isn’t large enough to avoid statistics bias, were conducted in our validation cohort. Thus, further studies should be conducted to verify the predictive efficacy of our signature.

In the study method, several studies had reported that LASSO-penalized regression could increase the accuracy of bioinformatic analysis and allow for simultaneous interpretation of each independent variable to select the most valuable parameters [30–33]. Because there were many confusing features, strong feature selection and shrinkage were still required to prevent overfitting, as well as increase interpretation. To address this issue, the least absolute shrinkage and selection operator (LASSO) Cox regression model, which is suitable for the regression of high-dimensional data [34, 35], was used for the further selection of prognostic mRNAs.

The upregulated CD4+ activated memory T cells, M2 macrophages, and neutrophils in the high stromal-score group may contribute to the poor outcome of PAAD [36]. In an investigation carried out by Tahkola K et al., immune scores were an independent prognostic factor for better disease-free survival and overall survival rates in PAAD patients [37], which may owe much to upregulated CD8 T lymphocytes, gamma delta T lymphocytes, resting NK cells, monocytes, and neutrophils in the high immune-score group [37]. Therefore, understanding the specific mechanism of how the portions of different types of immunocytes are regulated is essential for exploring novel therapeutic strategies to improve the immune response to PAAD. In this study, we revealed that three genes (CA9, CXCL9, and GIMAP7) from the 8-mRNA signature were responsible for the infiltration levels of immunocytes.

It is known that carbonic anhydrase IX (CA9) is a hypoxia-regulated, transmembrane protein associated with neoplastic growth in a broad spectrum of human tumours [38]. CA9 plays a direct role in stimulating an adaptive immune response, and the inhibition of CA9 reduces the capacity of cancer cells to acidify the extracellular environment, which could lead to enhanced immune activity [39, 40]. In stark contrast, CXCL9 is reported to suppress tumours by regulating immune cell migration, differentiation, and activation [41]. Moreover, the GTPases of the immunity-associated protein 7 (GIMAP7) are regulators of lymphocyte survival and homeostasis [42]. Consistent with these facts, our study confirmed that the expression levels of the three genes were related to the infiltration levels of several immune cells.

Forkhead box-O (FOXO) transcription factors have a fundamental role in the development and differentiation of immune cells, including dendritic Cells, T Cells, B Cells, and hematopoietic stem cells [43]. Mammals have 4 FOXO genes, FOXO1, FOXO3, FOXO4, and FOXO6, and these four genes are involved in multiple cellular pathways that regulate proliferation (FOXO1, FOXO3, and FOXO4), oxidative stress resistance (FOXO1 and FOXO3), metabolism (FOXO1 and FOXO3), cellular differentiation (FOXO3), inflammation (FOXO1, FOXO3, and FOXO4), aging (FOXO1, FOXO3, and FOXO4), and apoptosis (FOXO1, FOXO3, and FOXO4) in mammals [44]. Consistent with the results obtained by searching the TIMER web tool, the immune infiltration level in PAAD correlated with the expression levels of FOXO1 and FOXO3 but not FOXO4. Here, CA9 down-regulated, while CXCL9 and GIMAP7 up-regulated the expression of FOXO1 but not FOXO3 or FOXO4 in PAAD cells. Meanwhile, the IHC staining of PAAD patient specimens also revealed similar findings. Thus, our research demonstrated the critical effect of FoxO1 in regulating the immune response in PAAD.

## CONCLUSIONS

Applying ESTIMATE score to RNA-seq-based transcriptome and based on immune and stromal scores, we identified 333 DEGs between high and low stromal groups and 314 DEGs between high and low immune score groups, of which 203 genes were differentially expressed simultaneously that might regulate the immune response of PAAD patients, including immune cells infiltration level, participating in signalling pathways, and affecting prognosis. Furthermore, we used our results from the LASSO regression to construct a signature to evaluate each patient's risk based on the expression of the 8-mRNA and their regression coefficients, which can predict the prognoses of patients quite accurately, and we hope to apply it in clinical practice. Additionally, we validated that CA9, CXCL9, and GIMAP7 correlated with the OS of PAAD, and these three genes could specifically modulate the expression of FOXO1 to regulate immune infiltration in PAAD. Our investigation, therefore, produced new candidates for improving the immune response to PAAD.

## MATERIALS AND METHODS

### Downloading transcriptome datasets and the stromal and immune scores of PAAD

TCGA PAAD transcriptome FPKM data were downloaded from the GDC Data Portal (https://portal.gdc.cancer.gov/). Log2-transformed normalized ICGC PACA-Australia data were obtained from UCSC Xena (https://xenabrowser.net/). The included criteria for the analysis were: (a) pathological type was pancreatic carcinoma; (b) overall survival (OS) data were available; (c) raw count or normalized gene expression data were available. Per these criteria, we enrolled 176 and 69 pancreatic carcinoma cases for TCGA and UCSC, respectively [29]. Clinical data, such as the age, gender, race, survival time, and status of the selected patients, were also obtained from the two databases. We got the stromal and immune scores of TCGA PAAD patients from the ESTIMATE database (https://bioinformatics.mdanderson.org/estimate/index.html). The TCGA PAAD cohort was set as the training group for prognostic mRNA signature exploration, and the ICGC PACA group was used for validation. The mRNA names of probes were mapped according to their ensemble id, and the median value was used when several inquiries represented the same mRNA.

### Identifying differentially expressed mRNAs based on stromal and immune scores

We separated the TCGA cohort based on the median values of the stromal and immune scores of samples and obtained two groups of DEGs through the linear models for microarray data (LIMMA) R package. Fold change ≥2 and P <0.05 were set as the cut-off values for DEGs screening. Heatmaps were drawn using the heatmap R package. We also performed gene ontology (GO) enrichment analysis, KEGG pathway analysis, and protein-protein interaction enrichment analysis for DEGs using the Metascape (http://metascape.org) website tool [45].

### Estimating immune cell type proportions

The CIBERSORT R package with an LM22 signature and 1000 permutations was used to calculate the percentages of immune cells in PAAD cases. Per the description of a previous study [46], we inferred the proportions of immune cell types in the transcriptome data of cases and differences in immune cell type composition between different analysis groups (high immune score group vs low immune score group; high stromal score group vs low stromal score group) using the Microenvironment Cell Populations-counter method. To directly visualize the estimation result, we applied the “barplot” and “vioplot” R packages.

### Identifying prognosis-related genes

We selected overall survival (OS) as the endpoint and made verifications using the model for disease-free survival analysis. First, we used the univariate COX regression for the initial mRNA screening. The least absolute shrinkage and selection operator (LASSO) Cox regression model was then employed for the further selection of prognostic mRNAs [47]. We used the “Survival” and “glmnet” R packages for analyses and obtained the final model.

Based on the LASSO Cox regression result, we got a group of mRNAs and built an mRNA-based prognostic signature for the training cohort. Each patient’s risk score was calculated using a combination of the expression levels of mRNAs and LASSO-Cox regression coefficients. We used the median risk score as the cut-off value to divide the training set patients into a high-risk group and low-risk group, and the Kaplan-Meier curves and log-rank analysis were used to identify survival differences. The time-dependent receiver operating characteristic (ROC) curves of the risk scores were generated using the time ROC R package to explore the prognostic accuracy of risk scores. Univariate and multivariable Cox analyses were used to study the independent prognostic values of risk scores compared with other clinical characteristics. Lastly, the ICGC-AU data were used as the validation cohort to verify the effect of the risk-score model.

### Screening DEGs for further exploration

We further screened the genes in the LASSO Cox regression model for their biological regulatory mechanisms in PAAD. We used the webserver GEPIA (http://gepia.cancer-pku.cn/) to identify genes that could independently predict overall survival in patients with PAAD [18]. Considering the correlation between these genes and immune components, we also analyzed the relevance of interferon-gamma (as an essential immunoregulatory factor) to these genes. The web server TIMER (https://cistrome.shinyapps.io/timer/) was used to investigate the correlation between these genes and tumour-infiltrating immune cells [48].

### Immunohistochemistry (IHC)

To study altered protein expression, an IHC analysis was performed on tissue microarray (TMA) slides purchased from Outdo Biobank (Cat No. XT14-029) (Shanghai, China), using GIMAP7 antibody (Sigma, Cat# HPA020268,1:50), anti-CXCL9 antibody (Abcam, Cat# ab9720, 1:400), anti-CA9 antibody (Proteintech, Cat# 11071-1-AP, 1:200), and anti-FOXO1 antibody (Proteintech, Cat# 18592-1-AP, 1:1000). Semi-quantitative scoring was performed based upon the staining intensity (negative = 0; weak = 1; moderate = 2; and intense = 3). Two senior pathologists rated the degree of immunostaining of formalin-fixed, paraffin-embedded sections independently in a blinded manner. The scores were determined by the percentage of positive cells multiplied by the staining intensity. The clinical information on Tissue microarray (TMA) slides is shown in Supplementary Table 4.

### Statistical analysis

The R package was used to perform the differential expression genes (DEGs) analysis to obtain the genomic profile between high and low immune/stromal groups. The LASSO Cox regression model was employed for the further selection of prognostic mRNAs. The Chi-square test was used to calculate differences in clinicopathological variables between groups. The Kaplan-Meier method was used to determine disease-specific survival (DSS) and OS, and the differences between the study groups were compared using the log-rank test. Univariate and multivariate Cox proportional hazard regression models were used to calculate hazard ratios for OS and DSS. All the P values were adjusted using the FDR. A p-value less than 0.05 was considered significant. The described statistical analysis was performed with the IBM SPSS Statistics software version 24 for Windows (IBM Corporation, Armonk, NY, USA).

For RT-PCR and IHC analysis, statistical analysis was carried out using GraphPad Prism 6.0 (GraphPad Software Inc., San Diego, CA). Statistical significance was assessed using the unpaired two-tailed Student t-test between two groups or the one-way ANOVA with Tukey *post hoc* test for multiple comparisons. P values less than 0.05 were considered significant. All the values are expressed as the mean ± SD. Asterisks used to indicate significance correspond with *, P < 0.05; **, P < 0.01; ***, P <0.001.

## Supplementary Material

Supplementary Materials

Supplementary Figures

Supplementary Tables

Supplementary Table 1

Supplementary Table 5

Supplementary Table 6
