# Correction for: Using ESTIMATE algorithm to establish an 8-mRNA signature prognosis prediction system and identify immunocyte infiltration-related genes in Pancreatic adenocarcinoma

**DOI:** 10.18632/aging.205456

**Published:** 2023-12-28

**Authors:** Zibo Meng, Dianyun Ren, Kun Zhang, Jingyuan Zhao, Xin Jin, Heshui Wu

**Affiliations:** 1 Department of Pancreatic Surgery, Union Hospital, Tongji Medical College, Huazhong University of Science and Technology, Wuhan 430022, China; 2 Sino-German Laboratory of Personalized Medicine for Pancreatic Cancer, Union Hospital, Tongji Medical College, Huazhong University of Science and Technology, Wuhan 430022, China; 3 Department of Otorhinolaryngology-Head and Neck Surgery, Union Hospital, Tongji Medical College, Huazhong University of Science and Technology, Wuhan 430022, China; 4 Cancer Center, Union Hospital, Tongji Medical College, Huazhong University of Science and Technology, Wuhan 430022, China

**Keywords:** pancreatic cancer, tumor microenvironment, immunocytes infiltration, FOXO1

**This article has been corrected:** The authors recently found that the Kaplan-Meier survival curves in **Figure 4B**, which should have presented data for CXCL9, were an unintentional duplication of the survival curves in **4A**, which present data for CA9. The authors corrected the mistake. All data used in the study were from the TCGA public database and were generated using GEPIA online software. The authors apologize for any confusion or inconvenience caused by this error.

The corrected version of **Figure 4** is provided below.

**Figure 4:**
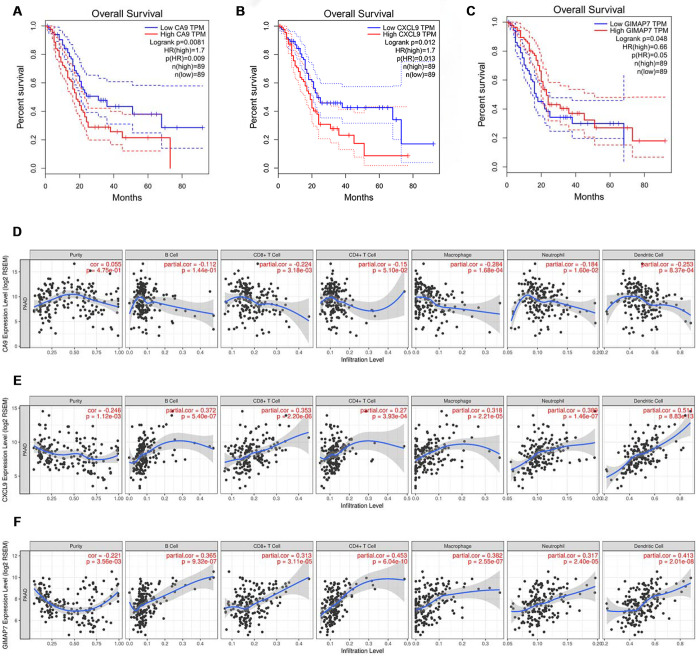
**CA9, CXCL9, and GIMAP7 regulate the immune infiltration of tumour microenvironment in PAAD. **(**A**–**C**) The overall survival rate of the patients with PAAD were computed with the GEPIA web tool. (**D**–**F**) The Timer web tool was used to determine the association between the expression levels of CA9 (**D**), CXCL9 (**E**) and GIMAP7 (**F**) with the infiltration level of immune cells in PAAD samples.

