# Supplementary material for: Using ESTIMATE algorithm to establish an 8-mRNA signature prognosis prediction system and identify immunocyte infiltration-related genes in Pancreatic adenocarcinoma
Source: Aging (Albany NY). 2020 Mar 17;12(6):5048–70. doi: 10.18632/aging.102931 (PMC7138590; doi:10.18632/aging.102931)
Supplement: Supplementary Materials [file aging-12-102931-s004..pdf]

SUPPLEMENTARY MATERIALS AND METHODS

Cell culture and RNA interference

PANC-1 and SW1990 cells were purchased from the Chinese Academy of Science Cell Bank. All cell lines were cultured in RPMI 1640 supplemented with 10% FBS. All cell lines were kept in a 37C incubator at 5% CO_2. The gene-specific siRNAs and control siRNA were purchased from RIBOBIO (Guangzhou, China). Pancreatic cancer cell line SW1990, PANC-1 and BxPC-3 were transfected with GIMAP7 siRNA, CXCL9 siRNA, CA9 siRNA or empty vector using Lipofectamine 2000 (Invitrogen Life Technologies) following the manufacturer's instruction. Six hours after transfection, the medium was replaced with fresh 1640 RPMI, containing 10% FBS. After 24–72 h of incubation in a humidified atmosphere (37°C and 5% CO_2), the experiments were performed. The sequence of siRNA, as indicated in Supplementary Table 2.

Western blotting analysis

Cells were washed for three times with phosphate buffer saline(KH_2PO^4 2mM, Na_2HPO^4 8mM, NaCl 136mM, KCL 2.6mM, pH7.2-7.4) on ice for more than 30 min and the cells were lysed on ice in prechilled lysis buffer (50mM Tris-HCl pH 7.5, 100mM NaCl, 1mM EDTA, 1% NP-40, 0.1% SDS, 20mM sodium fluoride, 2mM sodium orthovanadate, 20mM sodium pyrophosphate), containing a cocktail of protease inhibitors (Roche Diagnostics GmbH). The equal amounts of protein were subjected to SDS-PAGE analysis and transferred to PVDF membranes (Pierce Biotechnology, USA). Membranes were fixed in 5% not-fat milk at room temperature for 1 hr and incubated at 4°C overnight with primary antibodies: anti-GIMAP7 (Sigma, Cat# HPA020268), anti-CXCL9(Abcam, Cat# ab9720), anti-CA9 (Proteintech, Cat# 11071-1-AP), and anti-FOXO1(Proteintech, Cat# 18592-1-AP). The membranes were stripped and re-probed with an anti-GAPDH antibody (Proteintech, Cat# 10494-1-AP) as a control. After washing for more than three times with TBST, the membranes were incubated for 1 hr with goat anti-rabbit IgG. The protein bands were washed for three times and treated with ECL detection reagents and exposed to X-ray films.

RNA extraction and real-time reverse transcription PCR

Total cellular RNA was isolated from cells using the RNeasy isolation kit (Qiagen, Cat. # 74106). RNA samples (1 μg) were reverse-transcribed using PrimeScript™ RT reagent Kit (TaKaRa, Cat. # RR037A). Quantitative real-time PCR was performed using One-Step SYBR PrimeScript™ PLUS RT PCR Kit (TaKaRa, Cat. # RR096A).

The sequences of the primers used for qRT-PCR are listed in Supplementary Table 1. Relative quantities of each gene were calculated using the Ct method with GAPDH as a reference. Values represent the average of three technical replicates from at least three independent experiments. RNA extraction and real-time reverse transcription PCR. The sequences of RT-qPCR primers are indicated in Supplementary Table 3.
