# Supplementary material for: Using ESTIMATE algorithm to establish an 8-mRNA signature prognosis prediction system and identify immunocyte infiltration-related genes in Pancreatic adenocarcinoma
Source: Aging (Albany NY). 2020 Mar 17;12(6):5048–70. doi: 10.18632/aging.102931 (PMC7138590; doi:10.18632/aging.102931)
Supplement: Supplementary Figures [file aging-12-102931-s006..pdf]

SUPPLEMENTARY FIGURES

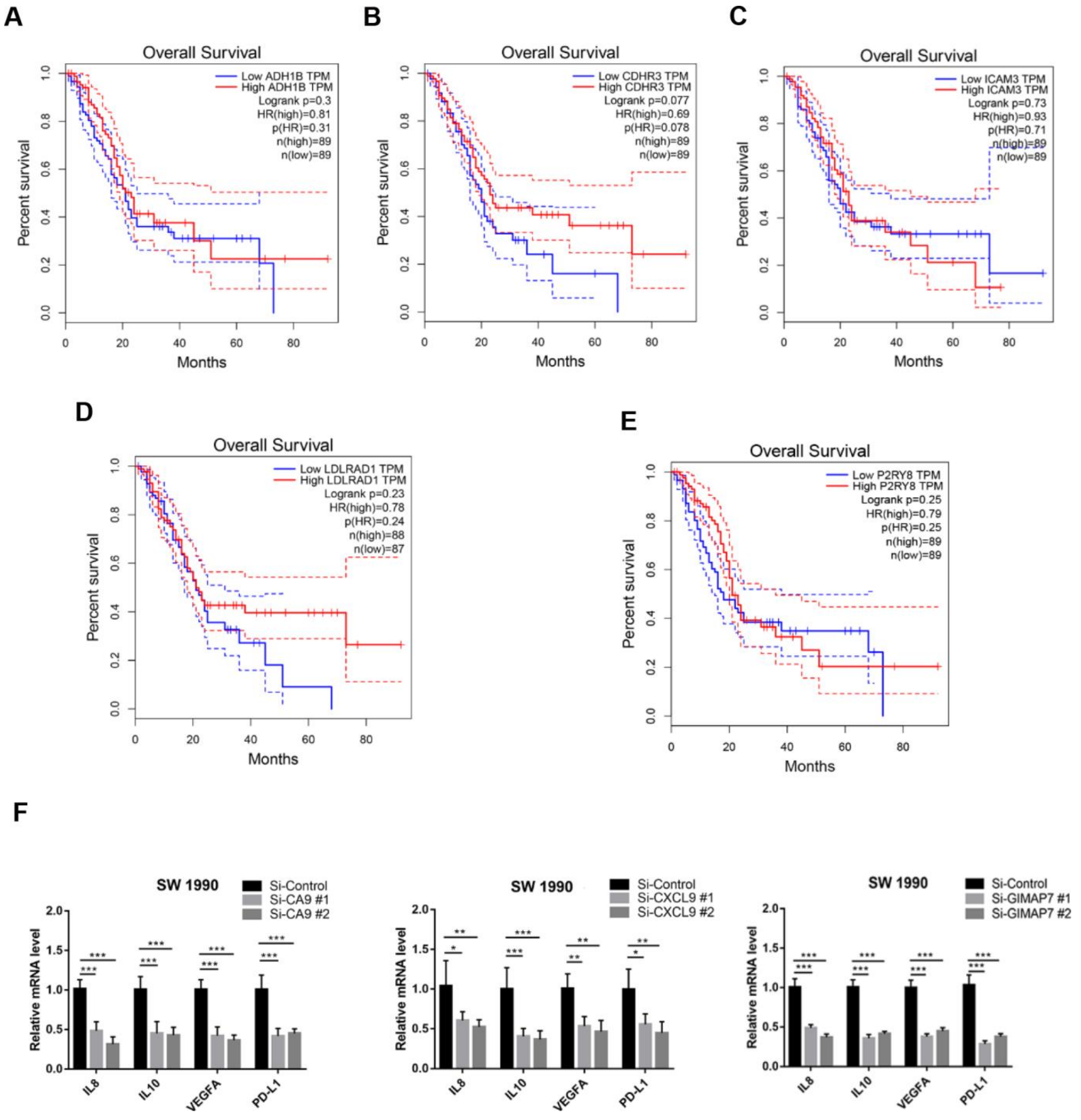

Supplementary Figure 1. (**A**–**E**) The overall survival (OS) of the patients with PAAD were computed with the GEPIA web tool. (**F**) SW 1990 cells transfected with indicated constructs. 48 h post-transfection, cells were harvested for RT-qPCR analysis. The data shown are the mean values ± SD from three replicates. *, P < 0.05; **, P < 0.01; ***, P < 0.001.

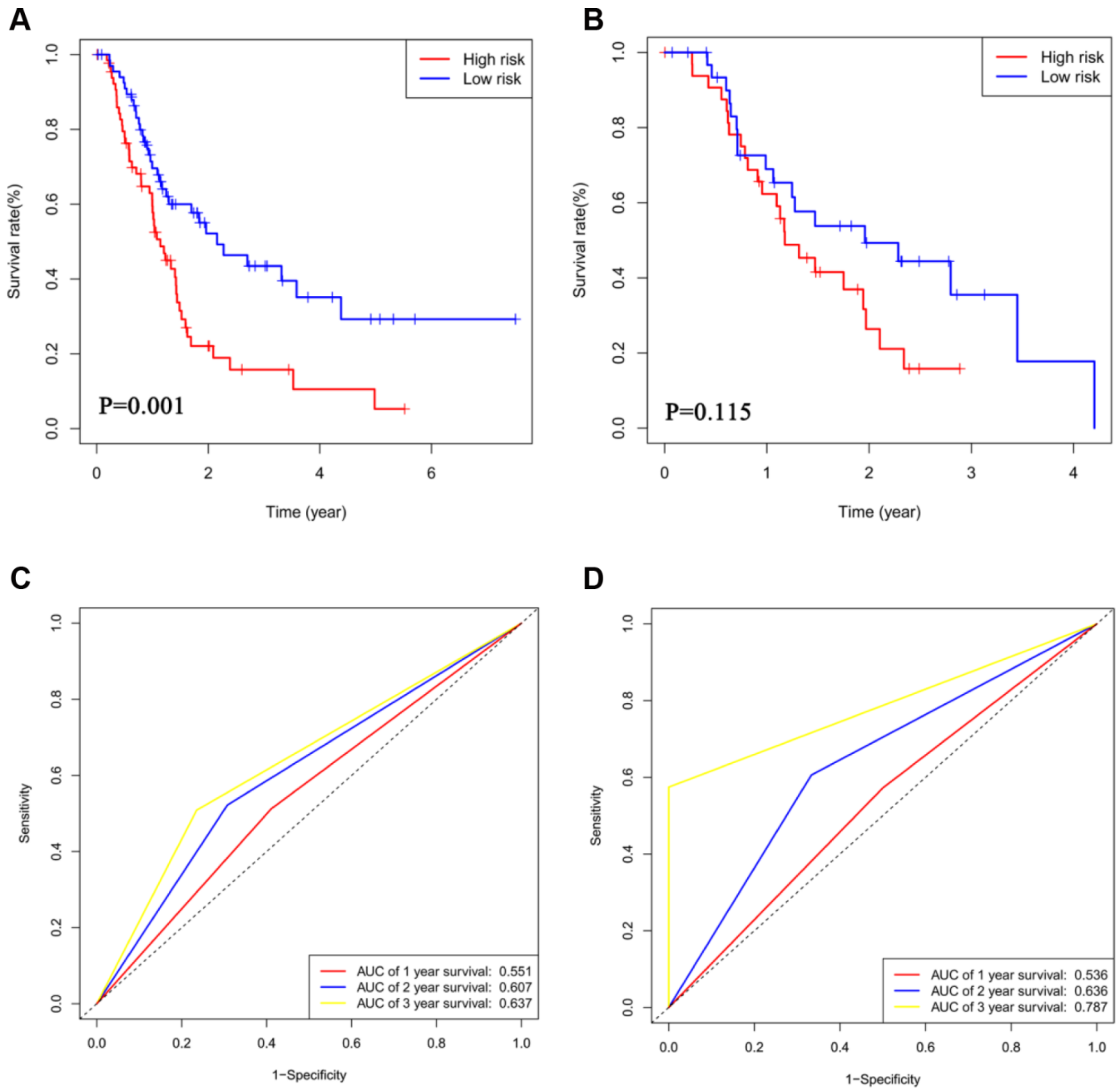

Supplementary Figure 2. (**A** and **B**) The Kaplan-Meier curves for DFS analysis in the training set (**A**) and the validation set (**B**). (**C** and **D**) Time-dependent ROC analysis for DFS analysis at 1, 2 and 3 years in the training set (**C**) and the validation set (**D**).
