# Supplementary material for: Using ESTIMATE algorithm to establish an 8-mRNA signature prognosis prediction system and identify immunocyte infiltration-related genes in Pancreatic adenocarcinoma
Source: Aging (Albany NY). 2020 Mar 17;12(6):5048–70. doi: 10.18632/aging.102931 (PMC7138590; doi:10.18632/aging.102931)
Supplement: Supplementary Tables [file aging-12-102931-s005..pdf]

SUPPLEMENTARY TABLES

Please browse Full Text version to see the data of Supplementary Table 1

Supplementary Table 1. Cox univariate regression for initial screening.

Supplementary Table 2. Sequences of gene-specific siRNAs.

siControl	Provided by RiboBio (Guangzhou, China)
siCA9-1	TTCAGCCGCTACTTCCAAT
siCA9-2	CAGCCGCTACTTCCAATAT
siCXCL9-1	CCAAGGGACTATCCACCTA
siCXCL9-2	GTTCGAAAATCTCAACGTT
siGIMAP7-1	GGCATAGGTTTTTGTCGAA
siGIMAP7-2	GACACCACCTGCAAGGAAA

Supplementary Table 3. Sequences of RT-qPCR primers.

Species	Gene	Forward (5′-3′)	Reverse (5′-3′)
Human	*GAPDH*	ACCCAGAAGACTGTGGATGG	TTCAGCTCAGGGATGACCTT
Human	*CXCL9*	CCAGTAGTGAGAAAGGGTCGC	AGGGCTTGGGGCAAATTGTT
Human	*CA9*	TTTGCCAGAGTTGACGAGGC	GCTCATAGGCACTGTTTTCTTCC
Human	*GIMAP7*	TCCAGGGCTCTTTGACACCA	AGAACTAGGACAATAGCATGGGG
Human	*FOXO3*	ACGGCTGACTGATATGGCAG	CGTGATGTTATCCAGCAGGTC
Human	*FOXO4*	CCGGCAAAAGCTCTTGGTG	GGTCCACATATCGGCTTCTTCA
Human	*FOXO1*	TCGTCATAATCTGTCCCTACACA	CGGCTTCGGCTCTTAGCAAA
Human	*IL-8*	ACTGAGAGTGATTGAGAGTGGAC	AACCCTCTGCACCCAGTTTTC
Human	*VEGFA*	AGGGCAGAATCATCACGAAGT	AGGGTCTCGATTGGATGGCA
Human	*IL-10*	GACTTTAAGGGTTACCTGGGTTG	TCACATGCGCCTTGATGTCTG
Human	*PD-L1*	GCTGCACTAATTGTCTATTGGGA	AATTCGCTTGTAGTCGGCACC

Supplementary Table 4. Clinical information of Tissue microarray (TMA) slides.

No.	Surgical organ	Sex(M/F)	Age	Pathological type	Pathological grading	Tumor size(cm)	Tumor location	T	N	M	AJCC 7th Cancer Staging Manual
1	pancreas	M	65	PDAC	II	2*2*2cm	pancreas head	T1	N0	M0	IA
2	pancreas	M	76	PDAC	II-III	1.5*1*1cm	pancreas head	T1	N0	M0	IA
3	pancreas	F	59	PDAC	II	2*2*1cm	pancreas head	T1	N0	M0	IA
4	pancreas	F	68	PDAC	II	3.5*2*2cm	pancreas head	T2	N0	M0	IB
5	pancreas	M	46	PDAC	II	8*6*4cm	pancreas body/tail	T2	N0	M0	IB
6	pancreas	M	74	PDAC	II-III	3.5*3*0cm	pancreas body	T2	N0	M0	IB
7	pancreas	M	50	PDAC	II	6*4*3cm	pancreas body/tail	T3	N0	M0	IIA
8	pancreas	M	53	PDAC	I-II	3.5*3.5*3cm	pancreas head	T3	N0	M0	IIA
9	pancreas	M	66	PDAC	II	3*2.5*2cm	pancreas head	T3	N0	M0	IIA
10	pancreas	F	75	PDAC	I-II	3*3*3cm	pancreas head	T3	N0	M0	IIA
11	pancreas	M	56	PDAC	III	4*3*3cm	pancreas body/tail	T2	N1	M0	IIB
12	pancreas	M	72	PDAC	I-II	5*5*4cm	pancreas head	T2	N1	M0	IIB
13	pancreas	M	64	PDAC	II-III	3*2*2cm	pancreas body	T2	N1	M0	IIB
14	pancreas	M	64	PDAC	II	4*2.5*2.5cm	pancreas head	T2	N1	M0	IIB
15	pancreas	M	72	PDAC	II- III	5*3*3cm	pancreas body/tail	T2	N1	M1	IV
16	pancreas	F	68	PDAC	II-III	3.5*2.5*1.5cm	pancreas head	T3	N1	M1	IV
17	pancreas	M	41	PDAC	II-III	4*3*3cm	pancreas body/tail	T3	N1	M1	IV
18	pancreas	M	59	PDAC	II-III	2.5*1.5*1cm	pancreas head	T3	N1	M1	IV
19	pancreas	M	63	PDAC	I-II	3.5*3*2.5cm	pancreas head	T2	N1	M1	IV
20	Metastases	F	71	PDAC	IV	——	liver Metastases	——	——	M1	IV
21	Metastases	——	——	PDAC	IV	——	liver Metastases	——	——	M1	IV
22	Metastases	M	74	PDAC	IV	——	liver Metastases	——	——	M1	IV
23	Metastases	M	47	PDAC	IV	——	abdominal soft tissue Metastases	——	——	M1	IV
24	pancreas	M	44	PDAC	II	3×1.5×1cm	pancreas head	T2	N1	M0	IIB
25	pancreas	F	52	PDAC	II	6×3×3cm	pancreas head	T2	N1	M0	IIB
26	pancreas	M	60	PDAC	II	4×3×3cm	pancreas head	T2	N1	M0	IIB
27	pancreas	M	62	PDAC	II	0.5×0.5×0.5cm	pancreas head	T1	N1	M0	IIB
28	pancreas	M	61	PDAC	II	7×4×2cm	pancreas head	T2	N1	M0	IIB
29	pancreas	M	52	PDAC	II	4×2×2cm	pancreas	T2	N1	M0	IIB
30	pancreas	M	63	PDAC	II	3.5×2.5×1.5cm	pancreas head	T2	N1	M0	IIB
31	pancreas	M	51	PDAC	II	5×4×3cm	pancreas body/tail	T2	N1	M0	IIB

Please browse Full Text version to see the data of Supplementary Tables 5, 6

Supplementary Table 5. DEGs between high and low stromal score groups.

Supplementary Table 6. DEGs between high and low immune score groups.
