# Supplementary material for: Using ESTIMATE algorithm to establish an 8-mRNA signature prognosis prediction system and identify immunocyte infiltration-related genes in Pancreatic adenocarcinoma
Source: Aging (Albany NY). 2020 Mar 17;12(6):5048–70. doi: 10.18632/aging.102931 (PMC7138590; doi:10.18632/aging.102931)
Supplement: Supplementary Table 1 [file aging-12-102931-s003..docx]

| **Supplementary Table 1.** **Cox univariate regression for initial screening** | | | | | | |
| --- | --- | --- | --- | --- | --- | --- |
| Genename | HR | P |  |  |  |  |
| CXCL9 | 1.176102 | 0.003944 |  |  |  |  |
| CDHR3 | 0.438903 | 0.00419 |  |  |  |  |
| LDLRAD1 | 0.595307 | 0.013826 |  |  |  |  |
| CA9 | 1.111891 | 0.019481 |  |  |  |  |
| P2RY8 | 0.749971 | 0.038765 |  |  |  |  |
| PPP1R16B | 0.748208 | 0.041261 |  |  |  |  |
| RASGRP2 | 0.739832 | 0.048533 |  |  |  |  |
| ICAM3 | 0.698758 | 0.063104 |  |  |  |  |
| TMEM190 | 0.628375 | 0.07288 |  |  |  |  |
| PLIN1 | 0.81686 | 0.073006 |  |  |  |  |
| CCR7 | 0.846694 | 0.077342 |  |  |  |  |
| GIMAP7 | 0.841885 | 0.079921 |  |  |  |  |
| ADH1B | 0.877864 | 0.08075 |  |  |  |  |
| TBC1D10C | 0.781932 | 0.084577 |  |  |  |  |
| TRARG1 | 0.731935 | 0.103295 |  |  |  |  |
| ADIPOQ | 0.838212 | 0.10719 |  |  |  |  |
| ZAP70 | 0.777774 | 0.10804 |  |  |  |  |
| TESPA1 | 0.711429 | 0.109583 |  |  |  |  |
| MAL | 0.77368 | 0.111362 |  |  |  |  |
| ACKR1 | 0.900797 | 0.113956 |  |  |  |  |
| GP1BA | 0.67787 | 0.1164 |  |  |  |  |
| LBP | 0.822404 | 0.118579 |  |  |  |  |
| ABCA8 | 0.803646 | 0.129728 |  |  |  |  |
| GZMM | 0.799395 | 0.131509 |  |  |  |  |
| CLEC17A | 0.720607 | 0.135927 |  |  |  |  |
| FABP4 | 0.914132 | 0.146093 |  |  |  |  |
| CIDEA | 0.759214 | 0.146853 |  |  |  |  |
| FCMR | 0.874577 | 0.152951 |  |  |  |  |
| CD163 | 1.115494 | 0.154245 |  |  |  |  |
| CD22 | 0.846998 | 0.162261 |  |  |  |  |
| CHRDL1 | 0.901512 | 0.163156 |  |  |  |  |
| NCR3 | 0.745742 | 0.169938 |  |  |  |  |
| LILRA4 | 0.731988 | 0.175007 |  |  |  |  |
| FAM129C | 0.779513 | 0.181822 |  |  |  |  |
| GPR18 | 0.737892 | 0.183292 |  |  |  |  |
| CMA1 | 0.765545 | 0.198681 |  |  |  |  |
| P2RX5 | 0.824719 | 0.203725 |  |  |  |  |
| P2RY13 | 0.829091 | 0.206089 |  |  |  |  |
| BTLA | 0.741477 | 0.209739 |  |  |  |  |
| PRKCB | 0.831218 | 0.210255 |  |  |  |  |
| MAP4K1 | 0.849055 | 0.210723 |  |  |  |  |
| CD5 | 0.863315 | 0.214415 |  |  |  |  |
| FCRL2 | 0.745982 | 0.219883 |  |  |  |  |
| AQP9 | 1.145558 | 0.220692 |  |  |  |  |
| PAX5 | 0.817563 | 0.229742 |  |  |  |  |
| CLEC10A | 0.882647 | 0.233653 |  |  |  |  |
| PLA2G2D | 0.859737 | 0.235265 |  |  |  |  |
| TRAF3IP3 | 0.81185 | 0.236572 |  |  |  |  |
| TLR10 | 0.793323 | 0.236997 |  |  |  |  |
| ARHGAP15 | 0.802942 | 0.238572 |  |  |  |  |
| HLA-DQA1 | 1.096996 | 0.249759 |  |  |  |  |
| CD79B | 0.907479 | 0.255836 |  |  |  |  |
| CD247 | 0.847052 | 0.260811 |  |  |  |  |
| TNFRSF13C | 0.874455 | 0.264222 |  |  |  |  |
| NTRK1 | 0.638328 | 0.265505 |  |  |  |  |
| FCRL3 | 0.807746 | 0.27084 |  |  |  |  |
| MPEG1 | 0.90281 | 0.27092 |  |  |  |  |
| RHOH | 0.845663 | 0.281812 |  |  |  |  |
| GAPT | 0.842198 | 0.283841 |  |  |  |  |
| PARP15 | 0.821976 | 0.286397 |  |  |  |  |
| LTA | 0.764344 | 0.2866 |  |  |  |  |
| KLRB1 | 0.888661 | 0.28963 |  |  |  |  |
| LEP | 0.85816 | 0.291416 |  |  |  |  |
| NCF1 | 0.835866 | 0.295266 |  |  |  |  |
| WDFY4 | 0.855285 | 0.300139 |  |  |  |  |
| CTSG | 0.879851 | 0.300165 |  |  |  |  |
| VPREB3 | 0.918527 | 0.303203 |  |  |  |  |
| EOMES | 0.783292 | 0.309439 |  |  |  |  |
| GPR183 | 1.088835 | 0.311095 |  |  |  |  |
| FCER2 | 0.891934 | 0.313891 |  |  |  |  |
| CLECL1 | 0.760958 | 0.324762 |  |  |  |  |
| IL16 | 0.875701 | 0.327097 |  |  |  |  |
| SCARA5 | 0.906161 | 0.33562 |  |  |  |  |
| EVI2A | 1.106844 | 0.339734 |  |  |  |  |
| AOAH | 0.8947 | 0.341339 |  |  |  |  |
| RIPOR2 | 0.869947 | 0.347867 |  |  |  |  |
| GZMK | 0.913962 | 0.361258 |  |  |  |  |
| ITK | 0.861839 | 0.365244 |  |  |  |  |
| C7 | 0.951931 | 0.367848 |  |  |  |  |
| CD27 | 0.914943 | 0.368971 |  |  |  |  |
| PI16 | 0.933598 | 0.37263 |  |  |  |  |
| BLK | 0.89078 | 0.3727 |  |  |  |  |
| IL24 | 0.888918 | 0.375331 |  |  |  |  |
| IRF4 | 0.868864 | 0.375453 |  |  |  |  |
| CD40LG | 0.839501 | 0.376241 |  |  |  |  |
| FPR2 | 1.161312 | 0.376829 |  |  |  |  |
| HLA-DOA | 1.082583 | 0.379033 |  |  |  |  |
| FCRLA | 0.894067 | 0.381047 |  |  |  |  |
| MS4A1 | 0.932005 | 0.388114 |  |  |  |  |
| HBB | 0.94939 | 0.391407 |  |  |  |  |
| PNCK | 0.888766 | 0.394337 |  |  |  |  |
| CSF3 | 0.871892 | 0.395463 |  |  |  |  |
| FCRL1 | 0.870816 | 0.395887 |  |  |  |  |
| SCIMP | 0.878392 | 0.39648 |  |  |  |  |
| CD28 | 0.852699 | 0.400174 |  |  |  |  |
| IKZF1 | 0.898568 | 0.40189 |  |  |  |  |
| PYHIN1 | 0.832925 | 0.402286 |  |  |  |  |
| SIRPG | 0.871739 | 0.404732 |  |  |  |  |
| CD19 | 0.915728 | 0.406074 |  |  |  |  |
| TRAT1 | 0.835266 | 0.411695 |  |  |  |  |
| PIK3R5 | 0.886612 | 0.415566 |  |  |  |  |
| RCSD1 | 0.91542 | 0.428054 |  |  |  |  |
| C3AR1 | 1.077993 | 0.428375 |  |  |  |  |
| SLAMF1 | 0.869798 | 0.434376 |  |  |  |  |
| CD37 | 0.932508 | 0.434724 |  |  |  |  |
| HAS1 | 0.918902 | 0.436273 |  |  |  |  |
| LRRC26 | 0.908983 | 0.439104 |  |  |  |  |
| MNDA | 1.076141 | 0.440841 |  |  |  |  |
| FCGR3B | 1.093497 | 0.442361 |  |  |  |  |
| SH2D1A | 0.895407 | 0.452333 |  |  |  |  |
| SIT1 | 0.917675 | 0.452995 |  |  |  |  |
| P2RY10 | 0.899884 | 0.4602 |  |  |  |  |
| GPR174 | 0.855582 | 0.461993 |  |  |  |  |
| FPR1 | 1.072826 | 0.464676 |  |  |  |  |
| TCL1A | 0.936969 | 0.466157 |  |  |  |  |
| CD1C | 0.931838 | 0.470362 |  |  |  |  |
| LILRB1 | 0.879879 | 0.471211 |  |  |  |  |
| CYTIP | 1.076638 | 0.471361 |  |  |  |  |
| ITGAL | 0.926246 | 0.479258 |  |  |  |  |
| CASS4 | 0.823787 | 0.480483 |  |  |  |  |
| CCL21 | 0.965055 | 0.483481 |  |  |  |  |
| PLA2G7 | 1.070051 | 0.488324 |  |  |  |  |
| CYBB | 1.058072 | 0.48917 |  |  |  |  |
| CCR4 | 0.907739 | 0.489314 |  |  |  |  |
| FPR3 | 1.061837 | 0.494123 |  |  |  |  |
| SAMSN1 | 1.082337 | 0.49491 |  |  |  |  |
| CCL19 | 0.969679 | 0.506325 |  |  |  |  |
| SIGLEC8 | 0.900615 | 0.519346 |  |  |  |  |
| TFF3 | 0.974336 | 0.534893 |  |  |  |  |
| STAP1 | 0.914603 | 0.536197 |  |  |  |  |
| TIGIT | 0.903524 | 0.542048 |  |  |  |  |
| TNFRSF17 | 0.92499 | 0.542866 |  |  |  |  |
| SASH3 | 0.94582 | 0.551892 |  |  |  |  |
| MEDAG | 1.04327 | 0.563731 |  |  |  |  |
| CLEC4E | 1.076802 | 0.569552 |  |  |  |  |
| MS4A7 | 0.946701 | 0.570026 |  |  |  |  |
| IKZF3 | 0.930287 | 0.572692 |  |  |  |  |
| CD3G | 0.913369 | 0.578439 |  |  |  |  |
| LAX1 | 0.912078 | 0.58433 |  |  |  |  |
| THRSP | 0.896523 | 0.58465 |  |  |  |  |
| RGS18 | 0.904814 | 0.58601 |  |  |  |  |
| CD48 | 0.952016 | 0.586559 |  |  |  |  |
| EVI2B | 1.048487 | 0.591344 |  |  |  |  |
| MS4A4A | 1.051122 | 0.596486 |  |  |  |  |
| TLR8 | 1.085097 | 0.597348 |  |  |  |  |
| CD53 | 1.041633 | 0.607354 |  |  |  |  |
| ICOS | 0.898677 | 0.611838 |  |  |  |  |
| IGF1 | 0.90949 | 0.612443 |  |  |  |  |
| ANGPTL7 | 0.945764 | 0.646783 |  |  |  |  |
| BTK | 0.944716 | 0.659841 |  |  |  |  |
| GPR171 | 0.94177 | 0.661935 |  |  |  |  |
| KLHL6 | 0.941374 | 0.675337 |  |  |  |  |
| FOLR2 | 0.966075 | 0.693445 |  |  |  |  |
| TNFSF8 | 0.940743 | 0.693494 |  |  |  |  |
| CMKLR1 | 0.953557 | 0.69436 |  |  |  |  |
| CD96 | 0.944976 | 0.701007 |  |  |  |  |
| RNASE6 | 0.965926 | 0.701928 |  |  |  |  |
| P2RY12 | 0.927519 | 0.704899 |  |  |  |  |
| NCKAP1L | 0.957098 | 0.705104 |  |  |  |  |
| PTX3 | 0.972277 | 0.720549 |  |  |  |  |
| CD79A | 0.979385 | 0.721751 |  |  |  |  |
| CD209 | 0.950704 | 0.743623 |  |  |  |  |
| CXCR4 | 1.025787 | 0.75079 |  |  |  |  |
| CCR2 | 0.955994 | 0.753793 |  |  |  |  |
| CXCL13 | 0.982773 | 0.75677 |  |  |  |  |
| CR2 | 0.982695 | 0.781843 |  |  |  |  |
| TIMD4 | 0.952908 | 0.782513 |  |  |  |  |
| CTLA4 | 0.959572 | 0.791632 |  |  |  |  |
| CD3E | 0.977516 | 0.794788 |  |  |  |  |
| CD180 | 0.963182 | 0.799848 |  |  |  |  |
| CSF2RB | 0.974195 | 0.802122 |  |  |  |  |
| DOCK2 | 0.969062 | 0.807568 |  |  |  |  |
| SELL | 0.98107 | 0.810247 |  |  |  |  |
| CD38 | 0.960863 | 0.816694 |  |  |  |  |
| TLR7 | 0.962483 | 0.824865 |  |  |  |  |
| IL21R | 0.958325 | 0.831038 |  |  |  |  |
| CD84 | 1.027104 | 0.834263 |  |  |  |  |
| SIGLEC7 | 1.038585 | 0.834934 |  |  |  |  |
| SIGLEC14 | 0.976982 | 0.847756 |  |  |  |  |
| LILRB4 | 1.023519 | 0.847822 |  |  |  |  |
| CXCR2 | 1.035996 | 0.850147 |  |  |  |  |
| CRTAM | 0.952034 | 0.854009 |  |  |  |  |
| LILRB2 | 1.027688 | 0.856636 |  |  |  |  |
| GPR34 | 0.982173 | 0.867572 |  |  |  |  |
| OGN | 0.989231 | 0.879124 |  |  |  |  |
| CXorf21 | 0.977097 | 0.880119 |  |  |  |  |
| CD52 | 1.010525 | 0.888441 |  |  |  |  |
| CD3D | 0.989421 | 0.902506 |  |  |  |  |
| JCHAIN | 1.004688 | 0.913693 |  |  |  |  |
| SLA | 1.009844 | 0.925783 |  |  |  |  |
| SP140 | 1.013517 | 0.931039 |  |  |  |  |
| CD8A | 0.991863 | 0.938695 |  |  |  |  |
| TFEC | 0.984267 | 0.940133 |  |  |  |  |
| TAGAP | 1.008862 | 0.942658 |  |  |  |  |
| PLEK | 1.006554 | 0.944878 |  |  |  |  |
| CD69 | 1.005856 | 0.954215 |  |  |  |  |
| IL7R | 1.004406 | 0.956863 |  |  |  |  |
| CCR5 | 0.993851 | 0.958776 |  |  |  |  |
| PTPRC | 1.003179 | 0.968838 |  |  |  |  |
| CD2 | 1.000941 | 0.991178 |  |  |  |  |
| CXCR6 | 1.001064 | 0.994456 |  |  |  |  |
| SLAMF6 | 0.999617 | 0.997111 |  |  |  |  |
| MS4A6A | 0.999714 | 0.99767 |  |  |  |  |
