# Supplementary material for: Using ESTIMATE algorithm to establish an 8-mRNA signature prognosis prediction system and identify immunocyte infiltration-related genes in Pancreatic adenocarcinoma
Source: Aging (Albany NY). 2020 Mar 17;12(6):5048–70. doi: 10.18632/aging.102931 (PMC7138590; doi:10.18632/aging.102931)
Supplement: Supplementary Table 5 [file aging-12-102931-s001..docx]

**Supplementary Table 5. DEGs between high and low stromal score groups.**

| **gene** | **log Fold change** | **P-Value** | **FDR** |
| --- | --- | --- | --- |
| ACKR1 | 1.5996885 | 1.42E-07 | 7.23E-07 |
| C1QTNF3 | 1.5212839 | 4.14E-12 | 4.83E-11 |
| TIMD4 | 2.05794 | 1.62E-09 | 1.19E-08 |
| ACTG2 | 1.8543192 | 7.19E-06 | 2.58E-05 |
| FNDC1 | 1.6320848 | 3.95E-15 | 8.65E-14 |
| PARP15 | 1.5531715 | 1.75E-07 | 8.73E-07 |
| LILRB5 | 1.7648027 | 8.89E-19 | 4.81E-17 |
| TMEM238 | -1.628698 | 6.20E-16 | 1.59E-14 |
| CPXM1 | 1.6376102 | 9.01E-17 | 2.76E-15 |
| XIRP1 | 1.9801644 | 3.10E-11 | 3.10E-10 |
| SIGLEC8 | 1.647204 | 1.13E-13 | 1.77E-12 |
| SULT1C2 | -1.646198 | 0.005432 | 0.009993 |
| BLK | 2.8584414 | 6.44E-06 | 2.33E-05 |
| CCDC80 | 2.006343 | 1.44E-26 | 6.16E-23 |
| NRK | 2.093576 | 5.09E-22 | 1.13E-19 |
| FIBIN | 1.6139857 | 1.56E-17 | 5.67E-16 |
| SLAMF6 | 1.5443177 | 4.55E-10 | 3.67E-09 |
| CD27 | 1.816926 | 1.42E-11 | 1.49E-10 |
| CLEC10A | 1.7712317 | 3.10E-12 | 3.70E-11 |
| EVI2A | 1.5975828 | 1.86E-22 | 5.16E-20 |
| PLA2G7 | 1.5062578 | 1.34E-16 | 3.98E-15 |
| ZAP70 | 1.6677546 | 5.29E-09 | 3.53E-08 |
| ADAMTS16 | 1.9396089 | 1.00E-15 | 2.45E-14 |
| GXYLT2 | 1.6368713 | 3.64E-23 | 1.65E-20 |
| LRRC26 | -4.425871 | 0.000195 | 0.000506 |
| GPR34 | 1.6282028 | 7.20E-19 | 4.06E-17 |
| GPR171 | 1.9056215 | 7.55E-14 | 1.24E-12 |
| OSR1 | 1.5640583 | 7.39E-10 | 5.75E-09 |
| COLEC12 | 2.0345621 | 3.04E-22 | 7.68E-20 |
| CCL19 | 1.9255663 | 1.27E-07 | 6.53E-07 |
| CTSG | 2.256215 | 3.30E-12 | 3.91E-11 |
| FMO1 | 1.7928106 | 7.63E-21 | 8.59E-19 |
| NTRK1 | 2.0691068 | 6.49E-10 | 5.10E-09 |
| KCNK2 | 1.5246194 | 2.25E-11 | 2.29E-10 |
| CA9 | -2.409528 | 1.58E-05 | 5.20E-05 |
| BTK | 1.6550753 | 1.67E-18 | 8.13E-17 |
| TRAT1 | 2.0928066 | 2.12E-12 | 2.61E-11 |
| PRRX1 | 1.5207483 | 2.56E-21 | 4.02E-19 |
| TLR10 | 2.2836298 | 4.51E-09 | 3.06E-08 |
| PNCK | -1.561633 | 1.77E-06 | 7.18E-06 |
| CCR2 | 1.7785165 | 2.08E-16 | 5.95E-15 |
| DPT | 1.7240478 | 1.82E-14 | 3.44E-13 |
| CCR1 | 1.6635265 | 9.34E-24 | 6.81E-21 |
| CRISPLD2 | 1.5231132 | 1.27E-21 | 2.27E-19 |
| RASSF2 | 1.6262417 | 1.35E-22 | 4.30E-20 |
| FABP4 | 2.157454 | 6.83E-07 | 3.02E-06 |
| PI16 | 2.4569889 | 1.03E-07 | 5.39E-07 |
| AOAH | 1.5123203 | 4.84E-17 | 1.57E-15 |
| AQP9 | 1.6093186 | 1.38E-14 | 2.68E-13 |
| CD19 | 2.6078788 | 2.76E-07 | 1.33E-06 |
| P2RY10 | 2.2683194 | 4.99E-15 | 1.06E-13 |
| NUDT10 | 1.6795791 | 5.77E-16 | 1.49E-14 |
| COL11A1 | 1.713221 | 6.75E-08 | 3.65E-07 |
| IL6 | 1.809632 | 7.95E-10 | 6.15E-09 |
| FBLN2 | 1.5206839 | 6.93E-23 | 2.45E-20 |
| SCIMP | 1.7556337 | 8.00E-19 | 4.45E-17 |
| CD52 | 1.5323801 | 5.09E-11 | 4.88E-10 |
| FCRL1 | 3.3106209 | 5.71E-07 | 2.57E-06 |
| FMO2 | 1.5759598 | 6.31E-14 | 1.06E-12 |
| HMGCS2 | -1.531213 | 0.000344 | 0.000844 |
| PAX5 | 2.4008016 | 0.000686 | 0.001561 |
| LDLRAD1 | -1.886727 | 0.004014 | 0.007635 |
| CXCL9 | 2.1482816 | 3.18E-10 | 2.63E-09 |
| BNC2 | 1.6644798 | 2.79E-23 | 1.44E-20 |
| GPR174 | 2.0823924 | 1.65E-12 | 2.08E-11 |
| APOBEC1 | -1.865531 | 0.001521 | 0.003182 |
| SCARA5 | 2.8613604 | 2.44E-10 | 2.07E-09 |
| CD2 | 1.6060737 | 5.52E-14 | 9.35E-13 |
| GFPT2 | 1.5512495 | 6.10E-21 | 7.27E-19 |
| CD79B | 2.1654465 | 6.33E-08 | 3.44E-07 |
| CD1C | 1.512448 | 3.78E-09 | 2.60E-08 |
| LAX1 | 2.0021294 | 5.87E-15 | 1.23E-13 |
| GPR183 | 1.6468282 | 1.30E-16 | 3.91E-15 |
| CCK | -1.655022 | 0.019574 | 0.031447 |
| CLECL1 | 1.6828925 | 1.88E-11 | 1.93E-10 |
| VPREB3 | 2.3472859 | 0.000413 | 0.000994 |
| ASPN | 1.5564159 | 4.59E-19 | 2.83E-17 |
| EVI2B | 1.6005891 | 2.77E-19 | 1.84E-17 |
| DOCK2 | 1.7849503 | 1.39E-22 | 4.32E-20 |
| ONECUT3 | -1.550917 | 4.53E-05 | 0.000135 |
| CLEC4E | 1.7435245 | 2.56E-14 | 4.69E-13 |
| EFEMP1 | 1.5667175 | 1.11E-19 | 8.42E-18 |
| CTLA4 | 1.5349034 | 1.68E-12 | 2.12E-11 |
| CMA1 | 2.6553941 | 3.11E-09 | 2.17E-08 |
| SIGLEC1 | 1.5304736 | 6.53E-17 | 2.05E-15 |
| SLA | 1.654323 | 1.03E-20 | 1.10E-18 |
| CSF2RB | 1.6229584 | 7.65E-18 | 3.02E-16 |
| DES | 2.1813993 | 2.40E-05 | 7.61E-05 |
| CD69 | 1.6646545 | 1.48E-12 | 1.89E-11 |
| F13A1 | 1.804078 | 2.71E-21 | 4.20E-19 |
| CCR7 | 2.3252669 | 3.99E-10 | 3.25E-09 |
| FEZF1 | -2.09495 | 0.000254 | 0.000643 |
| CXCL13 | 2.294534 | 1.85E-07 | 9.19E-07 |
| SYNM | 1.6176174 | 2.03E-05 | 6.54E-05 |
| CHRDL1 | 2.3215263 | 1.64E-13 | 2.51E-12 |
| FCMR | 2.1947031 | 1.46E-09 | 1.08E-08 |
| THBS1 | 1.7226512 | 3.20E-20 | 2.77E-18 |
| TNFSF8 | 2.3037105 | 3.80E-21 | 5.32E-19 |
| CASS4 | 1.5647571 | 7.42E-21 | 8.43E-19 |
| CD3D | 1.6032696 | 2.04E-11 | 2.08E-10 |
| ADH1B | 1.9904509 | 2.20E-08 | 1.30E-07 |
| TM4SF5 | -1.771852 | 6.93E-07 | 3.07E-06 |
| KLHL6 | 1.8282335 | 7.20E-19 | 4.06E-17 |
| RSPO3 | 1.7048549 | 9.55E-16 | 2.35E-14 |
| ALPK2 | 1.6275836 | 6.74E-13 | 9.22E-12 |
| HBB | 1.5605106 | 0.002595 | 0.005146 |
| TRAF3IP3 | 1.8539556 | 2.45E-14 | 4.50E-13 |
| JCHAIN | 1.9530222 | 1.22E-10 | 1.10E-09 |
| PLEK | 1.7697995 | 1.57E-20 | 1.55E-18 |
| MS4A6A | 1.5005369 | 1.34E-21 | 2.37E-19 |
| CELF2 | 1.5939187 | 1.14E-19 | 8.55E-18 |
| PTX3 | 2.258971 | 4.51E-09 | 3.06E-08 |
| HLA-DOA | 1.5028368 | 9.38E-18 | 3.63E-16 |
| P2RY8 | 1.5881934 | 1.39E-11 | 1.47E-10 |
| SPEF1 | -1.711666 | 0.006215 | 0.011266 |
| WT1 | 2.2161612 | 6.39E-22 | 1.34E-19 |
| OMD | 2.0195936 | 5.24E-19 | 3.19E-17 |
| CALHM3 | -1.624474 | 0.003903 | 0.007449 |
| SYNPO2 | 1.750568 | 1.68E-17 | 6.01E-16 |
| P2RX5 | 1.7474758 | 6.80E-06 | 2.45E-05 |
| SIGLEC14 | 1.5045664 | 1.39E-11 | 1.47E-10 |
| IL24 | 2.0568029 | 1.54E-09 | 1.13E-08 |
| FGF7 | 2.0028484 | 3.52E-19 | 2.25E-17 |
| CXCR4 | 1.5408396 | 4.93E-14 | 8.45E-13 |
| CSF3 | 1.8786394 | 0.016952 | 0.027671 |
| SH2D1A | 2.056015 | 1.10E-14 | 2.19E-13 |
| PLA2G2D | 2.130982 | 2.33E-08 | 1.38E-07 |
| MSR1 | 1.7007971 | 3.82E-22 | 9.29E-20 |
| IL7R | 1.9331873 | 2.59E-17 | 8.90E-16 |
| CD3E | 1.7634589 | 2.13E-13 | 3.18E-12 |
| CR2 | 2.4363969 | 3.63E-05 | 0.000111 |
| P2RY12 | 1.5034898 | 3.12E-10 | 2.59E-09 |
| MPEG1 | 1.8458281 | 4.75E-21 | 6.19E-19 |
| FOLR2 | 1.8118654 | 6.97E-22 | 1.42E-19 |
| IRF4 | 1.9472262 | 1.08E-13 | 1.70E-12 |
| P2RY13 | 1.5259354 | 2.09E-14 | 3.88E-13 |
| CD5 | 1.579687 | 2.04E-11 | 2.08E-10 |
| RHOH | 1.6837357 | 1.55E-12 | 1.97E-11 |
| TRARG1 | 2.613668 | 8.35E-06 | 2.95E-05 |
| CD28 | 2.0872882 | 7.03E-17 | 2.19E-15 |
| SAMSN1 | 1.5995626 | 2.87E-21 | 4.39E-19 |
| SCN7A | 1.770133 | 1.80E-10 | 1.57E-09 |
| CXorf21 | 1.5771272 | 4.36E-19 | 2.71E-17 |
| ARHGAP15 | 1.5384641 | 3.95E-15 | 8.65E-14 |
| CIDEA | 3.3304013 | 8.72E-05 | 0.000245 |
| SRPX | 1.694362 | 1.71E-18 | 8.31E-17 |
| GP1BA | 1.5886714 | 0.001132 | 0.002444 |
| WISP1 | 1.5710982 | 1.61E-20 | 1.58E-18 |
| CRTAM | 1.5414786 | 1.10E-15 | 2.66E-14 |
| SLC9A3 | -1.664017 | 0.027447 | 0.042501 |
| ICAM3 | 1.6858478 | 9.86E-14 | 1.57E-12 |
| SASH3 | 1.6969739 | 5.62E-18 | 2.33E-16 |
| MOXD1 | 1.6000381 | 1.95E-20 | 1.85E-18 |
| GAS1 | 1.9535047 | 3.43E-19 | 2.20E-17 |
| TFF3 | -2.14486 | 8.27E-05 | 0.000234 |
| CYTIP | 1.5896018 | 7.21E-17 | 2.24E-15 |
| MNDA | 1.6945309 | 4.05E-22 | 9.47E-20 |
| SIRPG | 1.6539862 | 7.09E-12 | 7.86E-11 |
| CLDN18 | -1.560851 | 0.014107 | 0.023436 |
| TRPM5 | -2.789615 | 0.003026 | 0.00591 |
| CD22 | 2.1791239 | 4.85E-09 | 3.27E-08 |
| SVEP1 | 1.7199788 | 1.92E-22 | 5.16E-20 |
| CXCR2 | 1.7905708 | 7.51E-13 | 1.02E-11 |
| MYH11 | 1.6413228 | 4.70E-11 | 4.54E-10 |
| RNASE6 | 1.5338222 | 3.54E-18 | 1.56E-16 |
| SFRP4 | 1.7682266 | 1.07E-18 | 5.54E-17 |
| RIPOR2 | 2.049294 | 2.74E-14 | 4.99E-13 |
| LBP | 1.9592635 | 1.97E-05 | 6.38E-05 |
| CILP2 | 1.7262464 | 6.19E-13 | 8.52E-12 |
| SIT1 | 1.9049492 | 4.79E-12 | 5.50E-11 |
| TMEM190 | -1.606643 | 0.00019 | 0.000497 |
| CDHR3 | -1.752735 | 8.79E-05 | 0.000247 |
| OGN | 2.2766476 | 6.60E-14 | 1.10E-12 |
| CD48 | 1.9819108 | 4.99E-13 | 6.97E-12 |
| PIK3R5 | 1.5638385 | 1.75E-20 | 1.68E-18 |
| MFAP4 | 1.5005844 | 2.52E-17 | 8.73E-16 |
| HSPB6 | 1.5059017 | 5.21E-13 | 7.25E-12 |
| CD38 | 1.636825 | 1.57E-15 | 3.67E-14 |
| CNN1 | 1.7118243 | 5.10E-13 | 7.11E-12 |
| FCRLA | 2.8297553 | 1.52E-08 | 9.32E-08 |
| TESPA1 | 1.7853674 | 5.90E-14 | 9.92E-13 |
| CD96 | 1.6145091 | 6.17E-14 | 1.03E-12 |
| KISS1 | -2.592846 | 6.63E-05 | 0.000191 |
| REG4 | -2.537589 | 0.009015 | 0.015732 |
| DCN | 1.5502087 | 1.05E-23 | 7.24E-21 |
| PDCD1LG2 | 1.6665836 | 4.47E-23 | 1.73E-20 |
| TFEC | 1.6997709 | 3.03E-21 | 4.53E-19 |
| LILRB2 | 1.5829751 | 6.54E-23 | 2.38E-20 |
| TNNI2 | -1.643083 | 0.005581 | 0.010235 |
| MUC5AC | -1.537786 | 0.000169 | 0.000446 |
| PLIN1 | 2.5923857 | 0.011285 | 0.019182 |
| FGF10 | 1.9417671 | 2.59E-18 | 1.18E-16 |
| IKZF1 | 1.9768875 | 6.53E-17 | 2.05E-15 |
| CD84 | 1.7085932 | 1.29E-20 | 1.34E-18 |
| SLAMF1 | 1.7171773 | 2.23E-13 | 3.31E-12 |
| CBLN4 | 1.7757323 | 4.64E-10 | 3.73E-09 |
| PTGER3 | 1.6344944 | 8.76E-21 | 9.69E-19 |
| FBN1 | 1.6570802 | 3.33E-23 | 1.65E-20 |
| TLR7 | 1.7768077 | 2.30E-19 | 1.57E-17 |
| RCSD1 | 1.8351701 | 4.35E-18 | 1.88E-16 |
| IKZF3 | 1.6870058 | 5.70E-10 | 4.53E-09 |
| TFF1 | -1.653931 | 3.07E-05 | 9.52E-05 |
| CHRDL2 | 1.8539059 | 5.78E-07 | 2.59E-06 |
| RUBCNL | 1.5835742 | 1.80E-18 | 8.65E-17 |
| DDR2 | 1.5096648 | 8.80E-24 | 6.81E-21 |
| FPR2 | 1.8170398 | 8.18E-13 | 1.09E-11 |
| HLA-DQA1 | 1.5352492 | 4.96E-17 | 1.60E-15 |
| CD247 | 1.5637911 | 6.01E-12 | 6.78E-11 |
| NPW | -3.235214 | 0.003112 | 0.006067 |
| GAPT | 1.8303579 | 1.03E-15 | 2.50E-14 |
| EPYC | 2.1148483 | 2.68E-06 | 1.05E-05 |
| FCRL2 | 2.5494415 | 3.92E-06 | 1.48E-05 |
| WDFY4 | 2.0125944 | 9.63E-18 | 3.72E-16 |
| LILRA4 | 1.9015901 | 5.42E-12 | 6.17E-11 |
| TFF2 | -1.592321 | 0.013266 | 0.022217 |
| ZIC2 | -1.910481 | 0.000151 | 0.000402 |
| CD163 | 1.901564 | 2.24E-20 | 2.10E-18 |
| TLR8 | 1.8613834 | 2.28E-18 | 1.06E-16 |
| SPON1 | 1.7235765 | 9.79E-21 | 1.05E-18 |
| TBC1D10C | 1.5584084 | 2.41E-08 | 1.42E-07 |
| FPR3 | 1.9454406 | 3.17E-26 | 9.82E-23 |
| CCR4 | 2.1356062 | 1.47E-15 | 3.43E-14 |
| SLIT3 | 1.8443964 | 2.86E-17 | 9.79E-16 |
| THRSP | 3.6989808 | 0.000473 | 0.001119 |
| MS4A4A | 1.6856948 | 3.04E-22 | 7.68E-20 |
| MAL | 1.5404106 | 1.05E-06 | 4.49E-06 |
| CD209 | 1.8660405 | 2.13E-16 | 6.08E-15 |
| LYVE1 | 1.6324012 | 8.63E-14 | 1.39E-12 |
| GPR18 | 1.8669055 | 3.86E-08 | 2.18E-07 |
| FPR1 | 1.6230834 | 9.01E-21 | 9.79E-19 |
| CD37 | 1.8383147 | 4.14E-15 | 9.03E-14 |
| CD53 | 1.6962239 | 3.80E-21 | 5.32E-19 |
| PDGFRL | 1.6592302 | 6.77E-22 | 1.40E-19 |
| SP140 | 1.705927 | 1.23E-12 | 1.60E-11 |
| CLMP | 1.6092102 | 1.54E-18 | 7.61E-17 |
| ICOS | 1.8286843 | 2.93E-14 | 5.28E-13 |
| SYNC | 1.6269027 | 1.42E-19 | 1.03E-17 |
| EBF2 | 1.7830644 | 2.18E-16 | 6.22E-15 |
| MAP4K1 | 1.666247 | 1.67E-11 | 1.72E-10 |
| CLEC17A | 2.6307132 | 2.23E-06 | 8.87E-06 |
| PHGR1 | -1.70261 | 0.000637 | 0.001461 |
| FCGR3B | 1.8020286 | 5.54E-12 | 6.28E-11 |
| GPNMB | 1.6804312 | 8.09E-26 | 1.72E-22 |
| TAGAP | 1.7207473 | 7.85E-18 | 3.08E-16 |
| C9orf24 | -1.554525 | 0.015425 | 0.025413 |
| FCER2 | 2.7462364 | 1.40E-05 | 4.67E-05 |
| SNORC | -1.789022 | 3.78E-11 | 3.73E-10 |
| ST6GAL2 | 1.6218714 | 1.07E-17 | 4.05E-16 |
| RASGRP2 | 1.8990245 | 9.74E-10 | 7.41E-09 |
| CD8A | 1.6000532 | 4.38E-13 | 6.19E-12 |
| CD40LG | 1.7632622 | 3.70E-10 | 3.03E-09 |
| SIGLEC7 | 1.5542043 | 6.45E-21 | 7.62E-19 |
| MEDAG | 2.3070215 | 4.60E-17 | 1.51E-15 |
| LILRB1 | 1.5806469 | 1.50E-19 | 1.07E-17 |
| NCF1 | 1.6871422 | 4.76E-15 | 1.02E-13 |
| RGS18 | 1.522193 | 5.11E-16 | 1.34E-14 |
| MMP3 | 2.1692078 | 0.00072 | 0.00163 |
| LRRC15 | 1.6588443 | 3.65E-07 | 1.70E-06 |
| IL21R | 1.6690536 | 1.18E-16 | 3.57E-15 |
| COL14A1 | 1.9294752 | 1.27E-16 | 3.83E-15 |
| CILP | 1.9192763 | 1.18E-15 | 2.82E-14 |
| HGF | 1.5061477 | 1.56E-17 | 5.67E-16 |
| NKX6-2 | -1.713625 | 0.007685 | 0.013598 |
| ITGA4 | 1.6272624 | 1.32E-18 | 6.66E-17 |
| PLIN4 | 1.9894614 | 0.007681 | 0.013592 |
| ADIPOQ | 3.4147498 | 4.08E-07 | 1.89E-06 |
| FCRL3 | 2.4453629 | 3.22E-09 | 2.24E-08 |
| EOMES | 1.9671756 | 6.60E-14 | 1.10E-12 |
| NCR3 | 2.0210329 | 2.07E-08 | 1.23E-07 |
| SLCO2B1 | 1.5226394 | 4.54E-22 | 1.02E-19 |
| HMCN1 | 1.5695584 | 5.68E-19 | 3.40E-17 |
| PPP1R16B | 1.5460233 | 7.04E-13 | 9.59E-12 |
| ABCA8 | 1.8387359 | 1.10E-14 | 2.19E-13 |
| MAB21L2 | 1.6643727 | 5.11E-09 | 3.42E-08 |
| SERPINF1 | 1.5171658 | 2.41E-22 | 6.36E-20 |
| TMEM74B | -1.56287 | 0.011476 | 0.019473 |
| SLIT2 | 1.5908954 | 8.06E-20 | 6.20E-18 |
| AMH | -1.776099 | 7.03E-08 | 3.79E-07 |
| FAM129C | 2.9424119 | 5.23E-05 | 0.000154 |
| BTLA | 2.3208671 | 7.81E-10 | 6.05E-09 |
| NR4A3 | 1.6427762 | 6.44E-11 | 6.03E-10 |
| FABP6 | -1.535583 | 0.005052 | 0.009362 |
| STAP1 | 2.2799808 | 1.60E-10 | 1.42E-09 |
| BOC | 1.5114163 | 6.85E-20 | 5.37E-18 |
| CXCR6 | 1.6723778 | 4.99E-15 | 1.06E-13 |
| ITGAL | 1.7324441 | 3.73E-16 | 1.01E-14 |
| ITK | 2.2027645 | 5.39E-14 | 9.17E-13 |
| BPIFA2 | -7.103014 | 0.010364 | 0.017827 |
| FBLN1 | 1.6442862 | 5.67E-20 | 4.53E-18 |
| GZMM | 1.5120346 | 1.26E-08 | 7.84E-08 |
| CD79A | 2.3563201 | 2.49E-08 | 1.47E-07 |
| KLRB1 | 1.5392212 | 7.53E-11 | 6.95E-10 |
| MFAP5 | 1.8047108 | 4.47E-19 | 2.77E-17 |
| PTPRC | 2.0918655 | 1.14E-19 | 8.55E-18 |
| TIGIT | 1.9430593 | 1.05E-15 | 2.56E-14 |
| CD180 | 1.6817739 | 8.36E-17 | 2.58E-15 |
| PDGFRA | 1.5130286 | 5.16E-21 | 6.53E-19 |
| CCL21 | 1.954022 | 4.33E-08 | 2.43E-07 |
| CYP1B1 | 1.7537259 | 1.12E-17 | 4.21E-16 |
| GIMAP7 | 1.5056565 | 1.47E-13 | 2.26E-12 |
| TNFRSF17 | 1.7989281 | 1.78E-09 | 1.29E-08 |
| CD3G | 1.793775 | 7.89E-14 | 1.28E-12 |
| LILRB4 | 1.5181932 | 3.62E-19 | 2.30E-17 |
| CYBB | 1.7619473 | 4.13E-21 | 5.56E-19 |
| CTGF | 1.6149615 | 4.02E-19 | 2.53E-17 |
| C7 | 2.1536758 | 1.63E-11 | 1.69E-10 |
| GZMK | 2.0171242 | 1.31E-12 | 1.69E-11 |
| MS4A1 | 2.8932561 | 3.19E-06 | 1.23E-05 |
| CCR5 | 1.8119873 | 4.20E-20 | 3.45E-18 |
| NTF4 | -1.661392 | 0.000244 | 0.000622 |
| MS4A7 | 1.7143226 | 4.34E-23 | 1.73E-20 |
| PYHIN1 | 2.0142568 | 1.32E-14 | 2.57E-13 |
| PNOC | 1.9859379 | 9.43E-14 | 1.51E-12 |
| ADAM12 | 1.8186493 | 1.21E-17 | 4.53E-16 |
| LTA | 1.69327 | 2.51E-12 | 3.06E-11 |
| LAMA2 | 1.5135029 | 8.21E-19 | 4.52E-17 |
| SFRP2 | 1.6232445 | 3.57E-20 | 3.05E-18 |
| SELL | 2.1005341 | 2.70E-11 | 2.71E-10 |
| TNNI3 | -1.535907 | 0.000835 | 0.001864 |
| PRKCB | 2.1406367 | 1.27E-15 | 3.00E-14 |
| ANGPTL7 | 1.7091148 | 1.39E-09 | 1.03E-08 |
| TNFRSF13C | 2.3877467 | 0.0002 | 0.000517 |
| CXCL12 | 2.003738 | 1.54E-19 | 1.08E-17 |
| LEP | 2.8441552 | 4.78E-09 | 3.23E-08 |
| TCL1A | 2.8073992 | 2.59E-07 | 1.26E-06 |
| C3AR1 | 1.570122 | 3.03E-21 | 4.53E-19 |
| IL16 | 1.6254711 | 2.87E-20 | 2.50E-18 |
| HAS1 | 2.7305098 | 1.01E-09 | 7.67E-09 |
| NCKAP1L | 1.6778385 | 2.17E-21 | 3.58E-19 |
| FAM180A | 1.6038473 | 1.57E-20 | 1.55E-18 |
| CMKLR1 | 1.5792846 | 7.04E-20 | 5.49E-18 |
| IGF1 | 2.8061645 | 5.49E-16 | 1.43E-14 |
| NEXN | 1.6024828 | 9.53E-22 | 1.82E-19 |
