# Supplementary material for: Using ESTIMATE algorithm to establish an 8-mRNA signature prognosis prediction system and identify immunocyte infiltration-related genes in Pancreatic adenocarcinoma
Source: Aging (Albany NY). 2020 Mar 17;12(6):5048–70. doi: 10.18632/aging.102931 (PMC7138590; doi:10.18632/aging.102931)
Supplement: Supplementary Table 6 [file aging-12-102931-s002..docx]

**Supplementary Table 6. DEGs between high and low immune score groups.**

| **gene** | **log Fold change** | **P-Value** | **FDR** |
| --- | --- | --- | --- |
| ACKR1 | 1.727874 | 1.35E-10 | 2.20E-09 |
| TIMD4 | 2.504156 | 2.18E-14 | 6.22E-13 |
| CCL23 | 2.074745 | 1.31E-17 | 5.91E-16 |
| PARP15 | 1.594457 | 1.78E-08 | 1.94E-07 |
| HCK | 1.511759 | 1.76E-25 | 1.15E-22 |
| TMC8 | 1.539871 | 2.65E-15 | 8.47E-14 |
| MCEMP1 | 1.97128 | 2.00E-11 | 3.74E-10 |
| FCN1 | 2.921544 | 7.21E-21 | 5.95E-19 |
| SIGLEC8 | 1.638326 | 3.16E-13 | 7.54E-12 |
| POU2AF1 | 1.69054 | 2.51E-07 | 2.22E-06 |
| BLK | 3.553623 | 4.72E-10 | 7.09E-09 |
| HTRA4 | 1.514257 | 4.21E-14 | 1.15E-12 |
| SLAMF6 | 1.90693 | 1.31E-17 | 5.91E-16 |
| CD27 | 2.18997 | 8.92E-18 | 4.11E-16 |
| CLEC10A | 1.960603 | 3.50E-17 | 1.46E-15 |
| EVI2A | 1.645961 | 5.97E-24 | 1.40E-21 |
| PLA2G7 | 1.612444 | 3.27E-18 | 1.60E-16 |
| ZAP70 | 2.069048 | 8.47E-16 | 2.91E-14 |
| MCOLN2 | 1.580273 | 5.11E-15 | 1.58E-13 |
| LILRA2 | 1.595635 | 9.26E-22 | 9.68E-20 |
| LRRC26 | -4.70001 | 0.00029 | 0.001212 |
| GPR34 | 1.527137 | 7.33E-16 | 2.55E-14 |
| CD8B | 1.70418 | 3.64E-16 | 1.32E-14 |
| GPR171 | 2.14341 | 1.31E-17 | 5.91E-16 |
| C1orf162 | 1.548572 | 4.99E-24 | 1.28E-21 |
| GPR65 | 1.637837 | 8.50E-23 | 1.24E-20 |
| CCL19 | 2.692612 | 6.30E-15 | 1.94E-13 |
| CEACAM21 | 1.649639 | 6.17E-23 | 9.58E-21 |
| CTSG | 1.880627 | 8.10E-10 | 1.17E-08 |
| CLEC12A | 1.804136 | 1.54E-19 | 9.41E-18 |
| NTRK1 | 1.995639 | 1.03E-07 | 9.70E-07 |
| PDE6G | 1.611165 | 1.93E-21 | 1.84E-19 |
| CA9 | -2.21725 | 1.82E-05 | 0.000106 |
| BTK | 1.950826 | 2.18E-26 | 4.28E-23 |
| TRAT1 | 2.361043 | 2.11E-17 | 9.10E-16 |
| LCP2 | 1.518652 | 5.82E-25 | 2.45E-22 |
| CHI3L2 | 1.50115 | 3.97E-12 | 8.33E-11 |
| CD72 | 1.621775 | 1.98E-16 | 7.54E-15 |
| ARHGAP30 | 1.548725 | 1.08E-23 | 2.13E-21 |
| CFP | 1.893782 | 2.12E-19 | 1.25E-17 |
| TLR10 | 2.691524 | 7.41E-15 | 2.26E-13 |
| PNCK | -1.9741 | 2.17E-06 | 1.56E-05 |
| CCR2 | 1.994843 | 1.83E-21 | 1.77E-19 |
| CORO1A | 1.89619 | 1.16E-20 | 9.03E-19 |
| DNASE1L3 | 1.653923 | 2.09E-09 | 2.78E-08 |
| FABP4 | 2.703561 | 9.50E-08 | 9.02E-07 |
| PI16 | 1.891477 | 2.58E-08 | 2.75E-07 |
| MADCAM1 | 1.536065 | 4.96E-07 | 4.15E-06 |
| AOAH | 1.966359 | 4.70E-24 | 1.28E-21 |
| AQP9 | 1.663134 | 5.11E-15 | 1.58E-13 |
| 1-Sep | 1.679801 | 1.76E-12 | 3.85E-11 |
| CD19 | 3.50601 | 4.14E-10 | 6.29E-09 |
| P2RY10 | 2.542355 | 9.23E-20 | 6.02E-18 |
| RASAL3 | 1.739928 | 5.08E-20 | 3.47E-18 |
| SPN | 1.759351 | 9.34E-24 | 1.90E-21 |
| CD6 | 1.71723 | 2.16E-18 | 1.09E-16 |
| ADAMDEC1 | 1.671678 | 4.41E-14 | 1.19E-12 |
| SCIMP | 2.026388 | 5.31E-25 | 2.32E-22 |
| CD52 | 2.168188 | 2.50E-24 | 8.19E-22 |
| FCRL1 | 3.970599 | 2.38E-10 | 3.75E-09 |
| GZMB | 1.586511 | 1.25E-12 | 2.80E-11 |
| PAX5 | 2.921894 | 6.33E-07 | 5.18E-06 |
| LDLRAD1 | -1.84787 | 0.005006 | 0.013657 |
| HCLS1 | 1.756272 | 5.07E-26 | 6.86E-23 |
| CXCL9 | 2.627786 | 6.35E-16 | 2.24E-14 |
| SRGN | 1.638298 | 3.70E-24 | 1.06E-21 |
| GPR174 | 2.355478 | 3.55E-16 | 1.29E-14 |
| SCARA5 | 2.307087 | 3.18E-10 | 4.92E-09 |
| CD2 | 2.001958 | 6.58E-22 | 7.06E-20 |
| CD79B | 2.971203 | 1.87E-13 | 4.63E-12 |
| CD1C | 1.945404 | 6.50E-16 | 2.29E-14 |
| LAX1 | 1.965738 | 1.83E-13 | 4.56E-12 |
| GPR183 | 1.770991 | 1.28E-19 | 8.05E-18 |
| CLECL1 | 2.145306 | 2.57E-20 | 1.87E-18 |
| VPREB3 | 3.334858 | 3.79E-08 | 3.89E-07 |
| CD4 | 1.561746 | 1.60E-25 | 1.11E-22 |
| EVI2B | 1.864503 | 1.14E-25 | 1.03E-22 |
| DOCK2 | 1.910821 | 3.95E-26 | 6.66E-23 |
| CLEC4E | 1.865035 | 3.01E-17 | 1.26E-15 |
| CTLA4 | 1.96296 | 7.42E-21 | 6.04E-19 |
| CMA1 | 2.302738 | 2.72E-08 | 2.89E-07 |
| SLA | 1.765902 | 4.99E-24 | 1.28E-21 |
| CSF2RB | 1.838473 | 2.48E-22 | 3.05E-20 |
| CD69 | 2.212663 | 1.50E-21 | 1.49E-19 |
| CCR7 | 2.917364 | 3.92E-18 | 1.88E-16 |
| CXCL13 | 3.204021 | 5.59E-12 | 1.14E-10 |
| CCL4 | 1.540693 | 2.01E-20 | 1.51E-18 |
| SHISAL2A | 1.862821 | 1.42E-18 | 7.41E-17 |
| CHRDL1 | 1.862752 | 3.81E-12 | 8.02E-11 |
| FCMR | 2.567293 | 3.51E-15 | 1.11E-13 |
| TNFRSF9 | 1.587303 | 3.00E-19 | 1.74E-17 |
| CASP14 | -2.77057 | 0.02379 | 0.049532 |
| MRC1 | 1.606124 | 9.23E-17 | 3.68E-15 |
| TNFSF8 | 2.320692 | 4.62E-21 | 3.95E-19 |
| DNAJC5B | 1.78354 | 4.13E-21 | 3.58E-19 |
| CASS4 | 1.502246 | 1.03E-19 | 6.59E-18 |
| EBI3 | 1.798432 | 3.72E-19 | 2.12E-17 |
| SIRPB1 | 1.995015 | 7.57E-17 | 3.03E-15 |
| IDO1 | 1.819179 | 1.83E-13 | 4.56E-12 |
| SUSD3 | 1.517872 | 1.07E-18 | 5.63E-17 |
| NGFR | 1.634769 | 1.13E-11 | 2.20E-10 |
| CD3D | 2.131684 | 3.80E-21 | 3.32E-19 |
| HLA-DOB | 1.799533 | 5.72E-11 | 9.92E-10 |
| ADH1B | 1.940506 | 3.00E-09 | 3.83E-08 |
| KLHL6 | 1.97318 | 4.81E-22 | 5.35E-20 |
| HBB | 1.514106 | 0.009289 | 0.0229 |
| TRAF3IP3 | 2.136744 | 9.01E-21 | 7.13E-19 |
| JCHAIN | 1.773085 | 1.48E-09 | 2.04E-08 |
| PDCD1 | 1.905522 | 2.41E-16 | 9.02E-15 |
| CD86 | 1.533862 | 1.17E-24 | 4.19E-22 |
| PLEK | 2.022452 | 8.70E-27 | 4.28E-23 |
| MS4A6A | 1.64761 | 1.25E-25 | 1.05E-22 |
| PARVG | 1.55336 | 2.14E-23 | 3.89E-21 |
| MYO1G | 1.537185 | 5.17E-23 | 8.60E-21 |
| KBTBD8 | 1.621321 | 1.82E-17 | 7.88E-16 |
| PTX3 | 2.037675 | 3.27E-07 | 2.83E-06 |
| LY86 | 1.708837 | 3.81E-24 | 1.07E-21 |
| RETN | 1.984343 | 7.64E-09 | 8.84E-08 |
| HLA-DOA | 1.577479 | 9.48E-20 | 6.11E-18 |
| P2RY8 | 1.842897 | 3.58E-17 | 1.49E-15 |
| CST7 | 1.571492 | 5.77E-18 | 2.71E-16 |
| CARMIL2 | 1.50159 | 9.10E-09 | 1.04E-07 |
| CD1D | 1.584063 | 2.77E-19 | 1.61E-17 |
| LTB | 2.009245 | 4.99E-16 | 1.78E-14 |
| P2RX5 | 2.178978 | 3.63E-11 | 6.53E-10 |
| SIGLEC14 | 1.98085 | 8.16E-18 | 3.78E-16 |
| IL24 | 2.239831 | 1.64E-13 | 4.14E-12 |
| CXCR4 | 1.735778 | 3.57E-20 | 2.51E-18 |
| POU2F2 | 1.605187 | 1.03E-13 | 2.67E-12 |
| CLEC7A | 1.573865 | 3.33E-23 | 5.87E-21 |
| CSF3 | 1.889336 | 0.003478 | 0.010186 |
| SH2D1A | 2.349295 | 1.21E-19 | 7.71E-18 |
| PLA2G2D | 3.053785 | 2.79E-17 | 1.18E-15 |
| SNX20 | 1.781395 | 9.20E-25 | 3.50E-22 |
| IL7R | 2.098845 | 4.36E-19 | 2.46E-17 |
| CD3E | 2.15564 | 3.12E-21 | 2.79E-19 |
| CR2 | 3.257205 | 3.17E-07 | 2.76E-06 |
| P2RY12 | 1.720737 | 2.23E-14 | 6.32E-13 |
| CCL5 | 1.730056 | 7.04E-20 | 4.67E-18 |
| MPEG1 | 1.878608 | 4.81E-22 | 5.35E-20 |
| FOLR2 | 1.650329 | 4.44E-20 | 3.08E-18 |
| LILRA5 | 1.680632 | 5.94E-21 | 5.00E-19 |
| CALHM6 | 1.690866 | 5.09E-17 | 2.08E-15 |
| IRF4 | 2.207888 | 4.24E-18 | 2.02E-16 |
| P2RY13 | 1.761553 | 2.18E-19 | 1.28E-17 |
| CD5 | 1.842082 | 1.60E-17 | 7.07E-16 |
| PPY | 3.129672 | 0.000885 | 0.00321 |
| LST1 | 1.508233 | 2.39E-25 | 1.49E-22 |
| RHOH | 1.973214 | 2.01E-19 | 1.19E-17 |
| TRARG1 | 2.822555 | 3.83E-05 | 0.000203 |
| CD28 | 2.194695 | 5.11E-19 | 2.84E-17 |
| SAMSN1 | 1.676776 | 2.56E-23 | 4.57E-21 |
| CXorf21 | 1.795637 | 5.46E-24 | 1.34E-21 |
| ACAP1 | 1.669147 | 9.34E-15 | 2.82E-13 |
| ARHGAP15 | 1.740099 | 1.03E-20 | 8.14E-19 |
| TNFAIP8L2 | 1.539493 | 6.58E-25 | 2.59E-22 |
| CIDEA | 3.42448 | 7.79E-05 | 0.000383 |
| GP1BA | 1.880755 | 7.70E-07 | 6.19E-06 |
| CRTAM | 1.812007 | 6.10E-21 | 5.07E-19 |
| ICAM3 | 2.225089 | 7.63E-21 | 6.16E-19 |
| SASH3 | 2.027725 | 2.70E-25 | 1.60E-22 |
| TFF3 | -2.01529 | 0.003555 | 0.010355 |
| CYTIP | 1.856264 | 4.70E-25 | 2.22E-22 |
| MNDA | 1.739998 | 2.02E-23 | 3.78E-21 |
| GBP5 | 1.964302 | 2.46E-18 | 1.23E-16 |
| SIRPG | 2.193457 | 1.83E-21 | 1.77E-19 |
| CD22 | 2.762671 | 1.79E-13 | 4.48E-12 |
| CXCR2 | 1.608386 | 4.06E-12 | 8.49E-11 |
| RNASE6 | 1.682494 | 1.97E-22 | 2.56E-20 |
| BIN2 | 1.674539 | 2.97E-25 | 1.67E-22 |
| SLAMF8 | 1.538169 | 9.83E-23 | 1.37E-20 |
| RIPOR2 | 2.273471 | 1.91E-17 | 8.26E-16 |
| WAS | 1.75486 | 5.23E-26 | 6.86E-23 |
| LBP | 1.999927 | 0.000142 | 0.000651 |
| SIT1 | 2.469315 | 2.71E-20 | 1.96E-18 |
| TMEM190 | -1.51694 | 0.002071 | 0.006599 |
| CD300LF | 1.628166 | 3.51E-22 | 4.10E-20 |
| CDHR3 | -1.70022 | 0.006673 | 0.017353 |
| OGN | 1.684769 | 1.25E-10 | 2.04E-09 |
| CD48 | 2.471485 | 9.83E-23 | 1.37E-20 |
| PIK3R5 | 1.71794 | 5.14E-24 | 1.29E-21 |
| CD38 | 1.961732 | 1.85E-18 | 9.49E-17 |
| SPIB | 1.995423 | 5.59E-10 | 8.30E-09 |
| FCRLA | 3.518793 | 8.95E-10 | 1.29E-08 |
| TESPA1 | 2.101039 | 3.34E-19 | 1.91E-17 |
| CD96 | 1.921579 | 3.03E-20 | 2.15E-18 |
| IGSF6 | 1.591929 | 2.82E-24 | 8.99E-22 |
| MARCO | 1.618371 | 5.88E-09 | 6.97E-08 |
| TFEC | 1.772437 | 9.28E-23 | 1.32E-20 |
| LILRB2 | 1.638491 | 1.59E-24 | 5.36E-22 |
| PLIN1 | 2.743581 | 0.003234 | 0.009588 |
| IKZF1 | 2.254924 | 2.21E-22 | 2.81E-20 |
| CD84 | 1.74525 | 2.34E-22 | 2.91E-20 |
| SLAMF1 | 2.1379 | 3.69E-21 | 3.25E-19 |
| CCL14 | 1.6104 | 9.39E-10 | 1.34E-08 |
| TLR7 | 1.720498 | 5.11E-19 | 2.84E-17 |
| RCSD1 | 1.975301 | 3.69E-21 | 3.25E-19 |
| DOK2 | 1.548434 | 1.60E-25 | 1.11E-22 |
| IKZF3 | 1.999168 | 1.86E-15 | 6.13E-14 |
| NLRP3 | 1.508069 | 1.30E-21 | 1.31E-19 |
| FPR2 | 2.162342 | 1.59E-14 | 4.62E-13 |
| HLA-DQA1 | 1.6807 | 6.40E-22 | 6.93E-20 |
| CD247 | 1.897712 | 1.31E-19 | 8.22E-18 |
| GAPT | 2.11627 | 8.06E-21 | 6.47E-19 |
| HP | 2.567917 | 0.003831 | 0.011009 |
| FCRL2 | 2.926073 | 2.04E-07 | 1.83E-06 |
| WDFY4 | 2.265841 | 2.08E-23 | 3.83E-21 |
| SIRPB2 | 1.630764 | 2.28E-22 | 2.86E-20 |
| LILRA4 | 2.265395 | 5.36E-16 | 1.90E-14 |
| IGLL5 | 1.615966 | 4.13E-09 | 5.11E-08 |
| CD163 | 1.889739 | 1.91E-19 | 1.14E-17 |
| LCP1 | 1.6188 | 3.04E-22 | 3.70E-20 |
| TLR8 | 2.198154 | 5.97E-24 | 1.40E-21 |
| TBC1D10C | 2.029152 | 1.98E-16 | 7.54E-15 |
| FPR3 | 1.757089 | 8.75E-22 | 9.22E-20 |
| CCR4 | 2.400103 | 6.15E-19 | 3.36E-17 |
| THRSP | 2.913396 | 0.000423 | 0.00169 |
| CXCL10 | 1.651809 | 3.49E-11 | 6.30E-10 |
| MS4A4A | 1.722778 | 4.09E-23 | 6.90E-21 |
| MAL | 1.708242 | 8.80E-11 | 1.48E-09 |
| CD209 | 1.58908 | 3.68E-13 | 8.75E-12 |
| GPR18 | 2.308503 | 4.31E-14 | 1.17E-12 |
| FPR1 | 1.678659 | 3.22E-22 | 3.84E-20 |
| CD37 | 2.191323 | 3.97E-23 | 6.80E-21 |
| CD53 | 1.95638 | 1.65E-28 | 1.95E-24 |
| SP140 | 2.176837 | 4.75E-21 | 4.03E-19 |
| ICOS | 2.254556 | 3.22E-22 | 3.84E-20 |
| MAP4K1 | 2.114138 | 1.26E-20 | 9.62E-19 |
| CLEC17A | 3.091546 | 4.22E-10 | 6.40E-09 |
| LAIR1 | 1.582942 | 5.93E-26 | 7.00E-23 |
| FCGR3B | 1.811918 | 3.99E-09 | 4.96E-08 |
| TAGAP | 1.897584 | 6.73E-23 | 1.03E-20 |
| FCER2 | 3.953469 | 5.07E-11 | 8.89E-10 |
| RASGRP2 | 2.218416 | 1.43E-15 | 4.77E-14 |
| CD1E | 1.807094 | 3.52E-14 | 9.69E-13 |
| CD8A | 1.827055 | 1.21E-16 | 4.80E-15 |
| CD40LG | 2.194218 | 6.36E-17 | 2.58E-15 |
| SIGLEC7 | 1.65867 | 1.59E-23 | 3.03E-21 |
| ARHGAP25 | 1.579688 | 5.82E-23 | 9.15E-21 |
| MEDAG | 1.6622 | 9.82E-08 | 9.27E-07 |
| LILRB1 | 1.788334 | 3.91E-25 | 2.01E-22 |
| NCF1 | 2.125973 | 1.10E-24 | 4.07E-22 |
| RGS18 | 1.749391 | 1.58E-19 | 9.62E-18 |
| CCL17 | 2.141513 | 1.75E-13 | 4.39E-12 |
| IL21R | 1.966789 | 6.22E-22 | 6.79E-20 |
| FYB1 | 1.610601 | 1.10E-18 | 5.75E-17 |
| HK3 | 1.534442 | 1.55E-16 | 6.00E-15 |
| ADIPOQ | 3.659123 | 3.65E-08 | 3.77E-07 |
| FAM78A | 1.664375 | 1.14E-22 | 1.51E-20 |
| CCL22 | 1.841687 | 1.64E-17 | 7.23E-16 |
| FCRL3 | 2.979291 | 2.19E-15 | 7.09E-14 |
| EOMES | 2.139814 | 5.34E-18 | 2.53E-16 |
| NCR3 | 2.389243 | 2.80E-14 | 7.84E-13 |
| PPP1R16B | 1.640925 | 2.23E-14 | 6.32E-13 |
| ABCA8 | 1.541961 | 2.34E-11 | 4.33E-10 |
| PTGDS | 1.512099 | 5.39E-10 | 8.03E-09 |
| IL10RA | 1.646961 | 4.84E-24 | 1.28E-21 |
| FAM129C | 3.674136 | 5.78E-09 | 6.88E-08 |
| IL2RB | 1.557282 | 1.04E-18 | 5.51E-17 |
| BTLA | 2.858508 | 1.24E-17 | 5.68E-16 |
| UBASH3A | 1.615575 | 6.66E-16 | 2.33E-14 |
| STAP1 | 2.850673 | 1.07E-14 | 3.22E-13 |
| CXCR6 | 1.903991 | 1.62E-19 | 9.83E-18 |
| ITGAL | 2.101517 | 1.49E-24 | 5.19E-22 |
| ITK | 2.468826 | 3.45E-18 | 1.67E-16 |
| KLK12 | -3.61925 | 0.017151 | 0.03793 |
| GZMM | 1.922239 | 1.38E-14 | 4.07E-13 |
| CD79A | 2.913232 | 2.74E-10 | 4.26E-09 |
| KLRB1 | 1.83948 | 1.13E-16 | 4.47E-15 |
| PTPRC | 2.261176 | 7.35E-23 | 1.11E-20 |
| TIGIT | 2.334234 | 1.01E-22 | 1.39E-20 |
| CD180 | 1.940285 | 8.50E-23 | 1.24E-20 |
| MZB1 | 1.558074 | 2.95E-08 | 3.12E-07 |
| NTS | 3.218692 | 0.021837 | 0.046265 |
| CCL21 | 1.92612 | 9.22E-10 | 1.32E-08 |
| GIMAP7 | 1.551589 | 1.23E-14 | 3.65E-13 |
| C1QB | 1.624612 | 7.36E-24 | 1.61E-21 |
| TNFRSF17 | 1.831795 | 1.16E-08 | 1.30E-07 |
| TNFSF18 | 1.593628 | 1.24E-09 | 1.72E-08 |
| CD3G | 2.08756 | 5.68E-19 | 3.12E-17 |
| LILRB4 | 1.7116 | 7.81E-24 | 1.68E-21 |
| CYBB | 1.928448 | 9.16E-26 | 9.01E-23 |
| C7 | 1.990118 | 3.56E-11 | 6.41E-10 |
| IL2RA | 1.785149 | 2.46E-18 | 1.23E-16 |
| GZMK | 2.422953 | 1.24E-19 | 7.88E-18 |
| MS4A1 | 3.691409 | 9.92E-10 | 1.41E-08 |
| CCR5 | 1.881752 | 2.36E-21 | 2.14E-19 |
| MS4A7 | 1.716553 | 3.41E-22 | 4.02E-20 |
| PTPN7 | 1.727449 | 5.24E-22 | 5.78E-20 |
| PYHIN1 | 2.245563 | 5.37E-20 | 3.64E-18 |
| LTA | 2.222063 | 6.67E-20 | 4.45E-18 |
| GMFG | 1.513492 | 1.92E-26 | 4.28E-23 |
| SELL | 2.374516 | 3.58E-17 | 1.49E-15 |
| JAML | 1.793464 | 6.58E-25 | 2.59E-22 |
| DPEP2 | 1.627758 | 9.28E-23 | 1.32E-20 |
| PRKCB | 2.11132 | 8.66E-19 | 4.66E-17 |
| ANGPTL7 | 1.73138 | 5.53E-08 | 5.52E-07 |
| FOXP3 | 1.645642 | 6.10E-21 | 5.07E-19 |
| TNFRSF13C | 2.920385 | 2.33E-08 | 2.51E-07 |
| VSIG4 | 1.742073 | 8.76E-21 | 6.99E-19 |
| LEP | 3.205364 | 1.28E-06 | 9.76E-06 |
| ARHGAP9 | 1.765382 | 1.04E-21 | 1.07E-19 |
| AIM2 | 1.701992 | 9.06E-12 | 1.80E-10 |
| SIGLEC10 | 1.773011 | 5.49E-23 | 8.99E-21 |
| OLR1 | 1.678696 | 6.66E-16 | 2.33E-14 |
| CCL18 | 1.56505 | 1.23E-12 | 2.76E-11 |
| TCL1A | 4.344166 | 1.17E-12 | 2.65E-11 |
| C3AR1 | 1.657227 | 8.29E-24 | 1.72E-21 |
| IL16 | 1.740299 | 6.67E-20 | 4.45E-18 |
| HAS1 | 2.304581 | 1.53E-06 | 1.15E-05 |
| NCKAP1L | 1.856789 | 1.80E-26 | 4.28E-23 |
| BCL2A1 | 1.526388 | 2.57E-20 | 1.87E-18 |
| CMKLR1 | 1.546083 | 4.20E-20 | 2.93E-18 |
| IGF1 | 2.199276 | 2.14E-10 | 3.39E-09 |
